# Effect of Stacking Sequence and Process Parameters on the Thermomechanical Response and Joint Characteristics of Multilayer Friction Stir Welds of AA2024/AA6061/AA7075

**DOI:** 10.3390/ma19214515

**Published:** 2026-10-27

**Authors:** Ana Gogorici, Daniela-Monica Iordache, Eduard Laurențiu Nițu

**Affiliations:** Faculty of Mechanics and Technology, National University of Science and Technology Politehnica Bucharest, 060042 Bucharest, Romania; daniela.c.iordache@upb.ro (D.-M.I.); eduard.l.nitu@upb.ro (E.L.N.)

**Keywords:** friction stir welding (FSW), dissimilar aluminum alloys, multilayer joint, stacking sequence, process parameters, thermomechanical response, material flow, microhardness

## Abstract

Friction stir welding (FSW) is an increasingly common solid-state joining process for aluminum alloys, including dissimilar alloy combinations. This study presents a detailed analysis of joining three plates made of different aluminum alloys, AA2024, AA6061, and AA7075, commonly used in the aeronautical industry, with a thickness of 2 mm, stacked on top of each other. The research was carried out in two stages. In the first stage, three different alloy overlap methods were analyzed while keeping the process parameters unchanged. In the second stage, a single overlap configuration was defined, and the two process parameters, welding speed and tool rotational speed, were varied. For the three first-stage and six second-stage experiments, the temperature and axial force developed during the process, surface roughness, macrostructure, microstructure, and microhardness distribution were evaluated. The results showed that both the stacking sequence and the process parameters influenced the thermomechanical response and the resulting joint characteristics. Among the investigated stacking sequences, AA7075–AA2024–AA6061 exhibited the fewest observed defects and was selected for the second experimental stage. For this configuration, 650 rpm and 100 mm/min produced the most favorable macrostructural outcome within the investigated parameter range.

## 1. Introduction

Aluminum alloys are extensively used in lightweight structures, particularly in the aerospace and automotive industries, owing to their high strength-to-weight ratio and favorable mechanical properties. Friction stir welding (FSW) provides an effective solid-state joining solution for these materials and has attracted considerable interest in applications requiring high-quality aluminum alloy joints. However, joining dissimilar aluminum alloys remains challenging because different material responses during FSW can affect the characteristics and quality of the resulting joint [1,2,3,4,5].

For alloys 2xxx, 6xxx, and 7xxx, FSW allows the formation of a stir zone—SZ characterized by dynamic recrystallization and accentuated grain refining [6,7]. The thermo-mechanically affected zone (TMAZ) and the thermally affected zone (HAZ) show distinct changes in microstructure and microhardness distribution, often with a “W” type profile in the case of precipitation-hardened alloys [6,8]. For AA2024, the relationship between microstructure, microhardness, and corrosion resistance is highlighted by correlating precipitation evolution with local hardness variations [9]. Recent studies show that optimizing process parameters simultaneously controls the evolution of dynamic recrystallization, precipitate distribution, and the microhardness profile in characteristic zones (SZ, TMAZ, HAZ) [10,11].

The initial metallurgical condition of the alloys is also an important factor governing microhardness evolution after FSW. For alloys processed in the annealed condition, such as AA6061-O, local increases in microhardness after FSW have been reported and associated with solid-state plastic deformation and microstructural changes within the processed region [12].

In designing lightweight structures, dissimilar joints are relevant because they allow the combination of alloys with different strengthening mechanisms and distinct thermomechanical responses, such as alloys from the 2xxx, 6xxx, and 7xxx series. Recent studies on dissimilar AA2024/AA7075 and AA6061/AA7075 FSW joints have shown that microstructural evolution, microhardness distribution, material flow, and defect formation are strongly influenced by material positioning and by the processing parameters controlling heat input and plastic deformation. In particular, for AA6061/AA7075 joints, the relationship among material mixing, welding defects, and local microhardness variations further demonstrates the importance of controlling the processing conditions when joining alloys with different thermomechanical responses [13]. Recent studies show that dissimilar joints, such as AA2024/AA7075 and AA6061/AA7075, exhibit distinct microstructural and textural developments at the interface, and that the final properties depend on the material arrangement (AS/RS) and the parameters controlling heat input and plastic flow. [3,7]. For AA2024/AA7075 structures, the literature indicates that microstructural evolution in the mixing zone (SZ) and the thermo-mechanically affected zone (TMAZ) depends strongly on initial compositional differences and the precipitation mechanisms specific to each alloy. [7,14]. EBSD studies on the 2024/7075 interface show significant changes in texture and degree of dynamic recrystallization depending on heat input and tool geometry [2].

Material positioning on the advancing or retreating side influences the flow shape and degree of mixing [15,16]. Investigations of plastic flow show that the distribution of material around the pin and the formation of lamellar structures or internal defects are controlled by the rotation-to-welding-speed ratio and the thermal-input window [17,18]. Control of the process parameters, particularly tool rotational speed, welding speed, and axial force, influences the thermomechanical input, material flow, weld-zone geometry, and defect formation. For dissimilar AA2024/AA7075 joints, appropriate combinations of these parameters have been associated with more favorable material flow around the pin, reduced tunnel formation, and the development of a finely recrystallized stir-zone structure [19]. These findings emphasize the need for a correlated assessment of processing parameters, thermomechanical response, and the resulting structural characteristics of the joint.

Friction stir welding of AA2024/AA7075 dissimilar joints shows that lamination direction and material combination relative to the welding direction directly influence flow behavior, dynamic recrystallization mechanisms, texture development, and microhardness distribution, causing significant variations in tensile strength and fracture zone location [20]. In AA2024/AA6061 welded structures, the combination of the FSW process with controlled welding media, such as underwater welding, and post-weld heat treatments reduces precipitate dissolution, limits abnormal grain growth in the mixing area, and allows the hardness and efficiency of the joint to be improved to values higher than those obtained by air welding [21]. FSW parameters directly control the balance among dynamic recrystallization, precipitate evolution, and microhardness distribution, and the optimal combination of rotational and feed speeds enables a homogeneous mixing zone with high mechanical strength and reduced internal defects [4,19,22].

The combination of AA2024, AA6061, and AA7075 in multilayer FSW joints is of interest because these alloys exhibit different mechanical and thermomechanical responses and are individually used in lightweight structural applications [1]. Integrating them into a multilayer configuration allows combining complementary material characteristics within the same structure and tailoring each alloy’s position to local structural requirements. Previous experimental work has demonstrated the feasibility of joining these three dissimilar alloys in an overlapping configuration by FSW [1]. AA2024 alloy is frequently used in fuselage panels because of its good fatigue resistance [23], while AA7075 is preferred in highly loaded structural areas such as stringers or stiffeners because of its high mechanical strength [6,24]. AA6061 offers a favorable compromise between weldability, cost, and mechanical performance and can be used in areas subjected to moderate or transitional loads [25,26]. From this perspective, dissimilar multilayer configurations are relevant to structural concepts such as metal sandwich panels and skin–stringer joints, in which material selection and positioning can be adapted to local functional requirements. In such structures, FSW enables the production of a continuous solid-state joint without rivets or adhesives [24].

For FSW trilayer joints, achieving the desired properties depends largely on the weld’s internal quality, as defects such as tunneling, kissing bonds, or porosity can become early sources of cracking under load. In a previous study, Nițu et al. (2022) analyzed an FSW trilayer joint composed of AA6061, AA7075, and AA2024, focusing on internal defect characterization and their effects on the joint’s mechanical response [27].

Earlier investigations on the same AA2024/AA6061/AA7075 trilayer system separately examined the influence of material positioning under fixed FSW conditions [28] and the effects of tool rotational speed and welding speed for a fixed stacking sequence [29]. A subsequent study by the authors [30] investigated the influence of rotational speed on AA2024/AA6061/AA7075 trilayer FSW joints, showing that increasing rotational speed favors macrostructural homogenization and a more uniform microhardness distribution, but can also promote defect formation and local variations in joint properties [30].

The authors also investigated the mechanical behavior of the same multilayer system. Tensile testing evaluated the influence of FSW process parameters on joint performance [31], while complementary investigations using digital image correlation (DIC) and acoustic emission (AE) analyzed strain localization and damage evolution during tensile loading [32]. These studies included the AA7075–AA2024–AA6061 stacking sequence processed at 650 rpm and 100 mm/min, corresponding to Experiment 4.3 in the present work.

Building on these previous investigations, the present study extends the experimental analysis through a sequential two-stage approach to evaluate, within a common experimental framework, the influence of stacking sequence and process parameters on the behavior of multilayer AA2024/AA6061/AA7075 joints. In the first stage, the three stacking configurations are re-evaluated under processing conditions different from those used in the previous material-position study [28]. Subsequently, the AA7075–AA2024–AA6061 configuration, which exhibited the most favorable macrostructural outcome among the investigated configurations, is examined within a redefined range of tool rotational speed and welding speed, established based on the findings of the previous process-parameter study [29].

In contrast to previous investigations, which separately addressed material positioning, process parameters, or mechanical behavior, the present study investigates, within a common experimental framework, the relationships among stacking sequence, process thermomechanical response, material flow, defect formation and localization, surface characteristics, and local microhardness response. This approach reveals that favorable variations in individual process or surface indicators do not necessarily correspond to improved macrostructural integrity. In particular, higher measured temperatures, lower axial forces, or lower surface roughness did not consistently coincide with fewer observed defects. Therefore, the present study demonstrates the need for a correlated process–structure–local property assessment, rather than reliance on a single indicator, to characterize and select processing conditions for multilayer FSW joints of dissimilar aluminum alloys. This integrated assessment is further supported by the quantitative characterization of defect number, area fraction, and maximum size across the six processing conditions, complemented by a spatial analysis of two contrasting conditions.

## 2. Materials and Methods

### 2.1. Base Materials

The investigated FSW joint consisted of three stacked aluminum alloy plates, each 2 mm thick, made of AA2024, AA6061, and AA7075. We investigated different stacking sequences, as described in Section 2.2.

The three aluminum alloys differ in chemical composition, mechanical properties, and metallurgical condition, leading to distinct responses during FSW. The AA2024-T3, AA6061-O, and AA7075-T6 aluminum alloy plates used in the experiments were supplied by AMAG Rolling GmbH (Ranshofen, Austria). Table 1 summarizes the chemical composition and mechanical properties of the materials. The reported values correspond to the material batches used in this study and were obtained from the manufacturer’s material certificates.

The three aluminum alloys used in the welded structure, AA2024, AA6061, and AA7075, differ in chemical composition, properties, and metallurgical condition. These alloys are widely used in industrial applications, particularly in the aeronautical field, due to their low weight and favorable combinations of mechanical strength, fatigue and corrosion resistance, and machinability.

### 2.2. Design of Experiments

The experimental plan for this study consisted of two consecutive stages. The first stage compared stacking sequences of the three aluminum alloys and selected one configuration for further investigation, while the second stage focused on a detailed analysis of the selected configuration. Accordingly, during the first stage, the positions of the three aluminum alloys within the welded structure were varied while keeping process parameters constant. The process parameters used in this first stage (600 rpm and 170 mm/min) were selected based on previous experimental investigations involving the same three aluminum alloys [1]. In the second stage, the selected configuration was retained, and the influence of the main FSW process parameters, namely the tool rotational speed (rpm) and welding speed (mm/min), was systematically evaluated. The investigated ranges (650–1100 rpm and 60–100 mm/min) were selected to provide distinct processing conditions for evaluating the effects of both parameters, while an intermediate condition (845 rpm and 80 mm/min) was repeated to assess process repeatability. Table 2 presents the experimental conditions used in both stages.

Experiments 4.5 and 4.6 were conducted under identical process conditions (845 rpm and 80 mm/min) to assess process repeatability at an intermediate parameter set.

The experimental design aimed to compare the effects of stacking sequence and the main process parameters within the investigated range. Experiments 4.5 and 4.6 were conducted independently under identical processing conditions (845 rpm and 80 mm/min) as a repeatability check under intermediate conditions. The other parameter combinations were not independently replicated; therefore, the study was not designed to establish the statistical significance of parameter effects. The observed trends are interpreted comparatively within the investigated experimental range.

### 2.3. Experimental Setup and Measurement Equipment

The FSW experiments were carried out using the experimental setup shown in Figure 1. The system comprised an FSW 4–10 welding machine (ISIM Timișoara, Timișoara, Romania), a workpiece positioning and clamping device, an FSW tool, and a data acquisition and monitoring system to record process parameters throughout the welding operation.

Because processing characteristics during FSW play an important role in determining weld bead characteristics, they were continuously monitored throughout the process. The recorded process characteristics during welding included the surface temperature in the joint area and the axial force applied along the FSW tool axis.

The temperature was monitored on the surface of the welded structure, in the weld-bead region immediately behind the FSW tool. The maximum temperature recorded within the monitored region of the thermographic image was used to evaluate the thermal evolution, rather than the temperature at a fixed geometric point. The axial force is the force acting along the tool axis during welding. Both the measured surface temperature and the axial force depend on the technological parameters, particularly welding speed and tool rotational speed, as well as on the stacking sequence of the three aluminum alloys. Both provide relevant information on the process’s thermal and mechanical response and affect weld-bead properties.

The surface temperature was recorded using a FLIR A40M infrared thermographic camera (FLIR Systems, Danderyd, Sweden), which has a measurement range of −40 to +2000 °C and an accuracy of ±2 °C [33]. The thermographic data were processed using ThermaCAM™ Researcher Pro 2.10 software (FLIR Systems, Danderyd, Sweden). The camera was positioned approximately 0.30 m (30 cm) from the monitored surface. For the measurements reported in this study, the radiometric parameters were set to an emissivity of ε = 0.50, a reflected temperature of 26 °C, an atmospheric temperature of 26 °C, and a relative humidity of 50%. Atmospheric transmission was set to 1.00, and the external optics temperature and transmission were set to 26 °C and 1.00, respectively. The temperature determined by infrared thermography represents the surface temperature within the monitored region and does not represent the internal material temperature or the maximum temperature developed within the stir zone. Consequently, the temperature values reported in this study are used for a comparative assessment of the surface thermal response under the different experimental conditions and not to characterize the through-thickness thermal field of the joint.

The axial process force was measured using a WIKA compression force transducer, model F1211 (WIKA Alexander Wiegand SE & Co. KG, Klingenberg, Germany), with a working range of 0–20 kN and a relative linearity error of ±0.3%, positioned along the primary axis of the FSW machine. The force signal was continuously recorded throughout the process, allowing the evolution of the axial force to be monitored during both the tool plunging phase and the welding phase. The recorded data were subsequently used to analyze the axial-force evolution and to determine the characteristic process indicators, including the maximum axial force during plunging, the mean and maximum axial forces during welding, and the stabilization distance.

### 2.4. FSW Tool Design

The FSW tool geometry is essential for heat generation, material flow, and weld quality [34]. It must be designed to generate sufficient heat and promote adequate material flow and mixing during welding. At the same time, the tool material must provide adequate mechanical strength, wear resistance, and thermal stability under the processing conditions. With this in mind, Figure 2 shows the geometric characteristics of the tool used in the experiments.

The welding tool pin shape and length were selected based on the total thickness of the three-plate stack. Accordingly, a non-tapered, threaded cylindrical pin with a standard M6 metric thread, a 1.0 mm pitch, a 60° thread profile, and a 5.3 mm length was used. The tool tilt angle was 0°, and the shoulder diameter was set to Ø22 mm to promote heat generation and material plasticization in the weld region. The tool was made of P20+S tool steel (40CrMnMoS8-6, material No. 1.2312), according to DIN 17350 [35], with a hardness of 42 HRC.

### 2.5. Preparation and Measurement of the Analyzed Samples

To characterize the welded joints, including macrostructural and microstructural analyses, microhardness measurements, and surface roughness evaluation, we defined the shape, dimensions, and position of the specimens. For each welded structure, we placed the first specimen at least 50 mm from the start of the weld bead and analyzed at least two specimens at different positions along the weld bead.

Figure 3 shows the shapes and positions of the specimens analyzed in the first-stage phase, and Figure 4 shows those analyzed in the second-stage experimental plan. We extracted the specimens from the welded structures using a water jet cutting machine (KMT Waterjet Systems GmbH, Bad Nauheim, Germany).

Sample preparation for macro- and microstructural analyses involved grinding, polishing, and chemical etching, whereas microhardness determination required only grinding and polishing. We performed grinding mechanically under continuous water cooling on a Minitech 263 dual-platen grinding machine (PRESI, Eybens, France) using silicon carbide metallographic papers with five different grit sizes. After grinding, we polished the surface with a textile material using an alumina-type polishing agent of fineness grade 3 μm (Al_2_O_3_). We chemically etched the polished surface by swabbing it with hydrofluoric acid (HF) for 15–20 s.

A portable digital roughness tester, MAHR PS-10 (Mahr GmbH, Göttingen, Germany), was used to measure the arithmetic mean surface roughness (Ra) of the weld bead. Measurements were taken at multiple positions along the weld bead, as shown in Figure 3 and Figure 4. At each position, three roughness measurements were taken at different points on the weld bead surface.

The macroscopic analysis was conducted using an OPTIKA ITALY metallographic microscope (OPTIKA S.r.l., Ponteranica, Italy), while the microscopic analysis was performed using an OLYMPUS BX51M microscope (Olympus Corporation, Hachioji, Tokyo, Japan) adapted for metallographic examination, capable of magnifications up to 500×.

For microhardness evaluation, measurements were performed using an INNOVATEST FALCON 500 microhardness tester (INNOVATEST Europe BV, Maastricht, The Netherlands). Vickers microhardness measurements were performed on the cross-section of the welded joints using a load of 2.94 N (300 gf) and a dwell time of 10 s.

The microhardness distribution of the tested samples was determined from measurements taken at 33 points along three lines, each corresponding to a different material in the welded structure. Each line had 11 measurement points spaced 1 mm apart and positioned symmetrically about the weld centerline. This distribution of microhardness points was chosen to assess the behavior of the three aluminum alloys across the characteristic regions of the FSW joint. Microhardness measurements of the base materials were performed under the same testing conditions on separate specimens from the same material batches.

### 2.6. Quantitative Assessment of Macrostructural Defects

To complement the macrostructural evaluation, we quantitatively analyzed cross-sectional images from the six second-stage experiments. Each image was individually calibrated using a known reference distance, enabling conversion of pixel dimensions to millimeters.

Defects were identified as dark regions associated with voids or material discontinuities. To minimize misidentification of dark regions that did not correspond to actual defects, the segmentation results were verified, and non-defect regions were excluded from the analysis.

To ensure consistent comparisons across all experimental conditions, we defined a common trapezoidal region of interest (ROI) within the weld cross-section. The analyzed region encompassed the weld-affected multilayer cross-section associated with the tool action and maintained the same geometric area across all experimental conditions. Using an identical ROI ensured that the quantitative defect indicators could be directly compared across the six second-stage experiments. We used a reference ROI area of 52.2 mm^2^ to normalize the detected defect area and calculate the defect area fraction.

The main steps of the image-processing and quantitative defect-assessment procedure are summarized in Figure 5.

For each experimental condition, the number of connected defect regions (*N_c_*), total defect area (*A_d_*), defect area fraction (*f_A_*), maximum individual defect area (*A_max_*), and maximum defect dimension (*L_max_*) were determined. The defect area fraction was calculated according to Equation (1):

(1) $$f_{A}=\frac{A_{d}}{A_{\mathrm{ROI}}}\times 100$$

where *A_d_* represents the cumulative area of the regions identified as defects and *A_ROI_* represents the reference area used for quantitative assessment.

In addition to the global quantitative defect assessment, we performed a zone-based analysis of two contrasting experimental conditions, Experiments 4.2 and 4.3. We selected these conditions because they exhibited markedly different defect responses across the investigated parameter range, with Experiment 4.2 representing a high-defect condition and Experiment 4.3 showing the lowest overall defect level. Additionally, the parameters used to make these two joints were at the minimum and maximum values in Table 2. For this analysis, we divided the reference region along the weld centerline and the nominal interfaces between the three stacked alloys, yielding six analysis zones: AA7075-R, AA7075-A, AA2024-R, AA2024-A, AA6061-R, and AA6061-A, where R and A denote the retreating and advancing sides, respectively. We then evaluated the distribution of detected defect regions and their cumulative area within each zone. Because this analysis aimed to characterize the spatial distribution of discontinuities rather than recalculate the total number of independent defects, the zone-specific counts were interpreted only as indicators of defect localization.

## 3. Results and Discussion

### 3.1. First-Stage Research Results

#### 3.1.1. Temperature Analysis of the First-Stage Experiments

One of the most important characteristics of the FSW process, recorded during joint formation, is the surface temperature in the welding area, which is strongly influenced by the process parameters and by the material at the top of the welded stack. Additionally, the thermal conditions developed during the process influence material softening and plastic flow, and consequently, the characteristics and quality of the FSW joint.

Figure 6 shows the results of the initial Design of Experiments, conducted to determine the selected configuration of the three alloys in the joined stack. In this graph, the two stages of the FSW process are differentiated: tool penetration into the materials to be joined and its advancement. A different evolution was observed for the three stacking configurations. The stacking configuration affected the measured surface temperature: the highest temperature was recorded when AA7075 was in the upper layer, followed by the configuration with AA6061 on top, while the lowest temperature was recorded when AA2024 was in the upper layer. These differences indicate that the material positioned in the upper layer influences the thermal response of the FSW process. In the second experiment, recordings stopped after 60 mm from the start of the weld bead, highlighting a process interruption due to surface defects on the bead’s upper side.

For all three stacking configurations, the maximum surface temperature recorded during FSW remained below the melting temperature reported for the corresponding alloy positioned in the upper layer.

#### 3.1.2. Axial Force Analysis of the First-Stage Experiments

The axial force developed during FSW is an important indicator of the process’s thermo-mechanical response and is strongly influenced by tool motion, material resistance to deformation, and process parameters. In the present experiments, we monitored axial force during both the tool penetration and welding phases. Figure 7 displays the evolution of the axial process force for the three initial experiments. The properties of the material in the upper layer of the joined stack can influence its resistance to deformation and, consequently, the axial force recorded during the process.

An important characteristic derived from the axial-force evolution is the longitudinal distance from the start of the weld bead to stabilization of the axial force/process (d_s_). This distance represents the length required for the process to reach a relatively stable axial-force regime and can be influenced by both the positions of the materials in the processed stack and the process parameters. In this experimental plan, the stacking sequence of the three materials is the variable, while the process parameters are kept constant. Another process characteristic that can influence the stabilization distance is the duration of tool retention during the penetration phase, given the faster temperature rise at the start of the process. A smaller value of d_s_ indicates that a relatively stable axial-force regime is reached over a shorter distance from the start of the weld bead. This characteristic can therefore provide information on process stabilization, although it should not be considered independently as a direct indicator of joint quality.

The characteristics that can be identified from the evolution of the axial force are: Fa max-p—the maximum axial force during the penetration phase; Fa max-s—the maximum axial force recorded during the welding phase; Fa med-s—the mean axial force within the stabilized welding region; and d_s_—the distance along the weld bead from its starting point to the point where the axial force reaches a relatively stable regime. Figure 8 shows the values of all characteristics listed above.

Experiment 3, in which AA7075 was positioned in the upper layer and the highest surface temperature was recorded along the weld bead, showed the most stable axial-force evolution among the three first-stage experiments. In Figure 8, the difference between the maximum forces in the two phases (penetration and welding) was smaller than in the other experiments, and the average force in the stabilized welding region was close to the maximum value. Additionally, the stabilization distance from the start of the weld bead was shorter than in the other two experiments.

#### 3.1.3. Macrostructure Analysis of the First-Stage Experiments

The macrostructure of the welded structure, a key feature, was examined using two samples from each of the three first-stage experiments. The two samples were positioned at 140 mm and 230 mm from the weld bead’s starting point. This rule could not be followed for structure 1 because defects during welding prevented the weld bead from being completed; for this structure, only the specimen positioned 140 mm from the start of the weld bead was analyzed.

Across all analyzed samples, the four characteristic zones of the FSW joint can be distinguished: the core or weld zone (SZ—Stir Zone), located at the center of the joint, where the greatest plastic deformation and the highest temperatures occur; the thermo-mechanically affected zone (TMAZ—Thermo-Mechanically Affected Zone), adjacent to the core and characterized by lower deformation and temperature; the heat-affected zone (HAZ—Heat-Affected Zone), where material changes are caused solely by the thermal cycle, without direct mechanical deformation induced by the tool; and the base material (BM—Base Material), located outside the HAZ, where the material’s structure and properties remain unaffected by the FSW process.

In the macrostructural images shown in Figure 9, the advancing side of the tool is labeled A, and the retreating side is labeled R. Identifying these two areas is important because it allows us to assess how the tool’s rotation direction affects the friction stir welding (FSW) process and the resulting joint’s macrostructural characteristics.

Using the same process parameters but varying the arrangement of the three materials, Experiment 3 (AA7075–AA2024–AA6061) produced the fewest defects. This result indicates that the stacking sequence influences material flow and defect formation within the trilayer joint. For the bottom alloy, the FSW tool pin penetrates only partially into this layer, resulting in less material displacement and mixing with the upper layers. Additionally, when AA7075 was positioned in the upper layer, burr-like defects were not observed.

Experiments with the first and second overlapping material variants show a high level of defects, as shown in Figure 9a,b, including cracks, channels, tunnels, and non-uniform stir zones. These defects are mostly observed at the interface between AA6061 and AA7075. Their occurrence at this interface indicates that the stacking sequence and local material flow conditions influenced joint formation. The largest tunnel width was 2.7 mm, observed at the AA6061-AA7075 interface in Experiment 1. In this experiment, the AA7075 alloy was placed at the bottom of the stack, and the welding tool pin did not fully penetrate this layer, limiting its direct interaction with the material and its contribution to material flow and mixing.

Another defect category, burrs, was observed in the first two experiments. Although an additional deburring operation can remove these surface defects, their occurrence further supports the influence of stacking sequence on material displacement at the weld surface. In Experiment 3, this defect type was not observed when the AA7075 alloy was positioned at the top of the experimental stack.

#### 3.1.4. Microstructure Analysis of the First-Stage Experiments

The microstructure of the FSW joint was analyzed only for Experiment 3, on the specimen located 140 mm from the start of the weld bead, in the regions affected by the combined thermal and mechanical effects of the process, namely the Stir Zone (SZ) and the Thermo-Mechanically Affected Zone (TMAZ), as shown in Figure 10.

For this structure, a well-defined material flow pattern is observed among the three aluminum alloys, with more homogeneous mixing in the upper region where AA7075 and AA2024 meet on both the advancing and retreating sides. The boundaries between materials are more noticeable at the interface between the lower materials, namely AA2024 and AA6061; these features indicate less material mixing at this interface under the investigated processing conditions.

The stir zone is more clearly defined on the advancing side, where material flow and mixing are more pronounced than on the retreating side. The boundaries between the three alloys remain visible in the SZ and TMAZ, and defects are minor, located in the upper part of the weld bead, with the largest pore measuring about 390 μm.

#### 3.1.5. Microhardness Analysis of the First-Stage Experiments

Microhardness is an important characteristic of the FSW joint. In the three experiments, microhardness was measured on specimens located 140 mm from the start of the weld bead, across three depth layers, with 11 points arranged symmetrically about the center of the weld bead. For comparison, microhardness was also measured under identical conditions for the three base materials before processing. No microhardness measurements were taken in areas with joint defects. Table 3 lists the recorded microhardness values for the base materials.

The microhardness values measured for Experiment 1 in the lower alloy (AA7075) are lower than those of the base material. In contrast, the values recorded for the upper alloy (AA2024) and middle alloy (AA6061) are generally comparable to those of the corresponding base materials (Figure 11). The observed microhardness distribution reflects local changes produced by the FSW process, including thermal and mechanical effects and the redistribution of the three materials within the joint.

The microhardness analysis of Experiment 2 shows that measured values vary across the welded joint, depending on local material distribution and regions affected by the FSW process. Greater variations in microhardness were observed in the stir zone, especially on the advancing side of the FSW tool, where material flow and mixing are more pronounced. The microhardness of the AA7075 alloy layer also varies significantly compared with the base material (Figure 12). These differences can be attributed to material redistribution during FSW and to the thermal and mechanical effects developed during the process.

A key difference in Experiment 3 compared with the previous ones is the availability of complete microhardness profiles, as measurements could be performed across the entire analyzed section because the joint had fewer defects.

The microhardness values in the profiles of the AA7075 and AA2024 alloys are lower than those of the base materials (Figure 13). By contrast, the microhardness values of the bottom layer, namely AA6061, are higher than those of the base material. These variations indicate that the three alloys respond differently to the thermal and mechanical conditions developed during FSW and to local material blending within the joint.

The microhardness values in the weld region are influenced by the local material distribution and by the thermal and mechanical effects of the FSW process. In experiments with few defects, complete microhardness profiles were observed, allowing a more thorough evaluation of microhardness variation across the joint. Structure 3 showed a more uniform microhardness distribution than the other first-stage configurations, along with fewer observed defects.

#### 3.1.6. First-Stage Research Conclusions

The first-stage results identified the most suitable material arrangement among the investigated configurations and formed the basis for the second-stage experimental plan. One main observation was that the stacking configuration affected the measured surface temperature. Experiment 3 recorded the highest temperatures, with AA7075 in the upper layer in contact with the FSW tool shoulder. Additionally, the stacking sequence and resulting thermo-mechanical conditions influenced the evolution of axial force. The axial force was more stable along the weld bead in Experiment 3.

At the macrostructural level, the fewest defects and more pronounced material mixing were observed on both the advancing and retreating sides of the tool in Experiment 3 (AA7075–AA2024–AA6061). At the microstructural level, material flow among the three aluminum alloys was clearly observed, while distinct boundaries between the materials remained visible in the SZ and TMAZ, indicating that complete homogenization was not achieved under the investigated conditions.

Microhardness values varied with material position and local thermal–mechanical conditions within the joint. Experiment 3 showed more complete and uniform microhardness profiles than the other first-stage configurations, along with fewer observed defects.

Taking these conclusions into account, the stacking sequence used in Experiment 3 (AA7075–AA2024–AA6061) was selected for the second experimental stage, during which the process parameters, namely welding speed and tool rotational speed, were varied.

### 3.2. Second-Stage Research Results

The second-stage analysis included monitoring surface temperature and axial force, evaluating surface roughness, conducting macrostructural examination, and measuring microhardness to compare the experimental conditions. A detailed microstructural analysis served a different purpose and was performed for Experiment 4.3 (650 rpm and 100 mm/min), selected based on the macrostructural evaluation as the condition with the fewest observed defects. Therefore, the microstructural observations are used for an in-depth characterization of this selected condition and not to establish systematic microstructural differences among all process-parameter combinations.

Based on the first-stage conclusions, which showed that Experiment 3 exhibited more pronounced material mixing and fewer defects than the other configurations investigated, the second-stage research focused on a single stacking sequence: AA7075–AA2024–AA6061. Maintaining the same stacking sequence allowed us to investigate the influence of process parameters independently of changes in material positioning. For this structure, the process parameters, namely the tool rotational speed (rpm) and welding speed (mm/min), were varied according to the experimental design shown in Table 2. The second experimental stage therefore evaluated how variations in these two parameters affect the process’s thermal and mechanical response and the resulting characteristics of the FSW joint. The analysis included surface temperature, axial force, surface roughness, macrostructure, microstructure, and microhardness, allowing correlation between process conditions and resulting joint characteristics.

#### 3.2.1. Temperature Analysis and Axial Force Analysis of the Second-Stage Experiments

Figure 14 and Figure 15 show the evolution of surface temperature and axial force recorded during the welding process for all six experiments in the experimental plan.

As in the previous section, these values were recorded throughout the process, including both the penetration phase and the formation of the weld bead along its entire length. Monitoring these characteristics provides information on the thermal and mechanical response of the materials under the different processing conditions. Since the stacking sequence was kept constant for all six experiments, differences in surface temperature and axial force can be analyzed in relation to variations in tool rotational speed and welding speed. Continuous recording also allows tracking of process evolution from tool penetration to the stabilized welding phase.

Figure 14 and Figure 16 show that the measured surface temperature increases with both tool rotational speed and welding speed, with tool rotational speed having a stronger influence within the investigated parameter range [31].

Increasing welding speed is associated with an increase in axial force, whereas increasing tool rotational speed is associated with a decrease in axial force recorded during welding. The highest axial force was observed in the experiment with the minimum tool rotational speed and the maximum welding speed.

Tool rotational speed had a stronger influence on the measured process response within the investigated parameter range; a 70% increase in tool rotational speed resulted in an approximately 7% increase in the measured surface temperature, while the maximum axial force decreased. The simultaneous increase in surface temperature and decrease in axial force with increasing rotational speed indicate changes in the material’s thermo-mechanical response under the investigated processing conditions.

An additional key aspect of the FSW process is the stabilization distance, referred to as ds. This feature depends on the time the tool is held at the end of the penetration phase, before starting the welding movement, because this dwell period influences the thermal conditions established before the welding phase begins. Consequently, d_s_ was used to compare the distance required to reach a relatively stable axial-force regime across the different combinations of tool rotational speed and welding speed.

#### 3.2.2. Surface Roughness Analysis of the Second-Stage Experiments

Surface roughness was evaluated to assess weld-surface quality and investigate the influence of the selected FSW process parameters. The weld bead surface condition is influenced by the interaction between the tool shoulder and the upper material layer during tool advancement. Therefore, roughness measurements were used to compare the surface characteristics across different combinations of tool rotational speed and welding speed.

For these six second-stage experiments, the arithmetic mean roughness (Ra) was measured on the weld bead surface where the material contacted the tool shoulder, at three positions along the weld bead: 80, 110, and 205 mm from the start of the weld bead. At each position, we took three measurements, as described in Section 2.5, and used the average Ra for comparison. Figure 17 shows the relationship between the recorded average roughness values and specimen position along the weld bead across all experiments in the second-stage experimental plan.

The results show that increasing the tool rotational speed and reducing the welding speed generally led to lower surface roughness. Roughness also tended to decrease along the weld bead under most of the investigated conditions. This trend indicates that the weld bead’s surface characteristics change as the process progresses; however, surface roughness should be considered primarily an indicator of surface condition, not a direct measure of the internal quality of the FSW joint.

#### 3.2.3. Macrostructure Analysis and Quantitative Defect Assessment of the Second-Stage Experiments

Figure 18 shows the macrostructures of the FSW joints at 220 mm from the start of the joint for the different combinations of tool rotational speed and welding speed examined in the second-stage experimental plan.

The macrostructural analysis of the FSW joints was conducted in one region of each weld bead, at 220 mm from the start of the joint. These positions were selected to evaluate the joint after the initial process region and farther along the weld bead. In all six experiments, the four characteristic regions of the FSW joint could be identified: SZ, TMAZ, HAZ, and BM. Macrostructural examination was also used to assess material distribution and identify the presence and location of defects under the different processing conditions.

To complement the visual assessment of the macrostructures and provide an objective comparison across the experimental conditions, we quantified defects in the cross-sections using the image-analysis methodology described in Section Materials and Methods. The analysis considered both overall defect content and individual defect size, as these metrics provide complementary information about joint integrity. Table 4 summarizes the results for the six experimental conditions.

The quantitative results confirm the differences observed in the macrostructural images. Experiment 4.3 (650 rpm and 100 mm/min) had the lowest defect content across all conditions investigated, with only 19 connected defect regions, a total defect area of 0.072 mm^2^, and a defect area fraction of 0.138%. This condition also produced the smallest individual defect, with *A_max_* = 0.022 mm^2^ and *L_max_* = 0.264 mm.

Increasing the tool rotational speed from 650 to 1100 rpm increased the defect content at both welding speeds investigated. At 60 mm/min, the defect content rose from 1.774% in Experiment 4.1 to 4.735% in Experiment 4.2, whereas at 100 mm/min it rose from 0.138% in Experiment 4.3 to 4.378% in Experiment 4.4. Welding speed was influenced by tool rotational speed. At 650 rpm, increasing the welding speed from 60 to 100 mm/min markedly reduced the defect area fraction from 1.774% to 0.138%. In contrast, at 1100 rpm, the same increase in welding speed produced only a modest reduction, from 4.735% to 4.378%. Within the investigated parameter range, increasing welding speed had a much stronger beneficial effect at the lower rotational speed.

In Figure 19, bubble size indicates the defect area fraction f_A_, while bubble color indicates the maximum defect dimension *L_max_*. Experiments 4.5 and 4.6 were conducted under identical process conditions and are slightly offset in the figure for visualization.

Experiments 4.5 and 4.6, conducted with the same nominal process parameters (845 rpm and 80 mm/min), exhibited defect area fractions of 3.230% and 2.785%, respectively. Although the overall defect content was of the same order of magnitude, the morphology and size of the detected discontinuities differed. In particular, Experiment 4.6 had fewer connected defect regions than Experiment 4.5 (40 vs. 67) but showed substantially larger maximum individual defect area and maximum defect dimension. These differences indicate variability in local defect formation even under nominally identical processing conditions and further support using multiple quantitative descriptors to evaluate joint integrity.

Quantitative indicators show that the total defect area fraction alone does not capture severity. Experiment 4.6 had a lower fraction (2.785%) than Experiments 4.2 and 4.4, yet it had the largest individual defect (*L_max_* = 2.268 mm) and the area (*A_max_* = 1.124 mm^2^). Assessing both overall defect content and the dominant defect size therefore better characterizes the joint’s macrostructural integrity.

To further examine whether differences in overall defect content were also associated with differences in defect localization within the multilayer joint, we selected Experiments 4.2 and 4.3 for a more detailed spatial comparison. These two conditions provide a particularly informative contrast, since Experiment 4.2 exhibited one of the highest defect area fractions (*f_A_* = 4.735%), whereas Experiment 4.3 exhibited the lowest (*f_A_* = 0.138%). Figure 20 shows the spatial distribution of detected defect regions across the three alloy layers and on the advancing and retreating sides.

Figure 21 provides a more detailed comparison of the spatial characteristics of defect formation in Experiments 4.2 and 4.3 by combining the cumulative defect area within each of the six analysis zones (Figure 21a) with the location and size of the largest individual defect detected for each condition (Figure 21b). This representation allows assessment of both the preferential regions of defect accumulation and the magnitude of the dominant discontinuity, complementing the global quantitative indicators reported in Table 4.

The quantitative zone-based results further confirm the markedly different defect distributions between the two selected conditions. In Experiment 4.2, the AA7075 layer showed the largest cumulative defect areas, reaching 1.386 mm^2^ on the advancing side and 0.925 mm^2^ on the retreating side. AA2024 showed considerably smaller values, while no defect area was detected in the analyzed AA6061 zones. The largest individual defect identified for this condition was *A_max_* = 0.805 mm^2^; *L_max_* = 1.930 mm, and it was also located in the upper region of the multilayer cross-section, confirming that the high overall defect content was primarily associated with localized discontinuity formation in the AA7075 layer.

A markedly different spatial distribution was observed in Experiment 4.3. We detected no defect regions in the analyzed AA7075 layer or on the joint’s retreating side. The remaining discontinuities were confined to the advancing side, with cumulative defect areas of approximately 0.061 mm^2^ in AA2024 and 0.064 mm^2^ in AA6061. Moreover, the largest individual defect was substantially smaller than that observed in Experiment 4.2 (*A_max_* = 0.022 mm^2^; *L_max_* = 0.264 mm). Thus, the reduction in overall defect content was accompanied by a pronounced change in defect localization, from extensive defect accumulation in the upper AA7075 region in Experiment 4.2 to limited, localized discontinuities in the middle-to-lower region of the advancing side in Experiment 4.3.

The contrasting behavior of Experiments 4.2 and 4.3 indicates that the processing parameters affected not only the overall quantity and size of detected defects but also their spatial distribution within the multilayer joint. Experiment 4.2 used the highest rotational speed (1100 rpm) and the lowest welding speed (60 mm/min), whereas Experiment 4.3 used the lowest rotational speed (650 rpm) and the highest welding speed (100 mm/min). Taken together with the thermal and axial-force responses discussed in Section 3.2.1, these results indicate that increased thermal input alone did not improve material consolidation. Instead, within the investigated parameter range, defect formation appears to depend on the balance among heat generation, tool-induced deformation, material transport, and interactions between the dissimilar alloy layers.

Among all investigated conditions, Experiment 4.3 (650 rpm and 100 mm/min) exhibited the lowest overall defect level across the main quantitative indicators. The additional zone-based analysis further showed that the remaining discontinuities were spatially confined to the advancing side rather than distributed throughout the multilayer cross-section. Therefore, within the investigated parameter range, this condition provided the most favorable macrostructural integrity and was selected for the detailed microstructural analysis presented in the following section.

The mechanical significance of this condition is further discussed in the Discussion section by comparing it with previously published tensile, DIC, and AE results for the same stacking sequence and processing parameters [31,32].

#### 3.2.4. Microstructure Analysis of the Second-Stage Experiments

Based on the quantitative defect assessment in Section 3.2.3, we selected Experiment 4.3 (650 rpm and 100 mm/min) for detailed microstructural examination because it showed the best macrostructural integrity among the conditions tested. The analysis focused on the characteristic regions of the FSW joint, material flow, and the interfaces among the three aluminum alloys.

This condition was selected based on the comparative macrostructural assessment of all six experiments because it exhibited the fewest observed defects. Therefore, the microstructural analysis was intended to provide a detailed characterization of this selected condition rather than a systematic comparison of microstructures across all process-parameter combinations. The analysis focused on the characteristic regions of the FSW joint, the material flow, and the interfaces between the three aluminum alloys. Figure 22 shows key microstructural features.

The flow among the three welded alloys is well-developed, but visible boundaries persist between the materials. The persistence of these boundaries indicates that the investigated processing conditions did not fully homogenize the three materials. As expected for a solid-state joining process, the materials undergo thermomechanical softening and plastic flow during FSW without reaching the melting state.

In the stir zone, a finer, more equiaxed grain structure is observed, consistent with the intense plastic deformation and thermal exposure characteristic of this region. In the thermo-mechanically affected zone, the grains exhibit a more deformed morphology due to the combined thermal and mechanical effects of the process. In the heat-affected zone, the material is affected predominantly by the thermal cycle, without direct mechanical deformation induced by the tool.

The defect is located on the advancing side, at the interface between the middle material (AA2024) and the lower one (AA6061). As in the first-stage experiments, defects are primarily observed on the advancing side of the FSW tool. Their distribution may be influenced by the shape of the threaded cylindrical pin and its rotation direction. The tool geometry and rotation promote material displacement between the different layers and between the advancing and retreating sides of the joint [32]. The largest pore measured approximately 216.5 μm, which is smaller than the maximum pore size observed in the other analyzed experiments.

#### 3.2.5. Microhardness Analysis of the Second-Stage Experiments

Microhardness was measured in two regions of each weld bead, at 65 and 220 mm from the start of the joint, to assess variation along the weld bead and across the three material layers. The measurements also allowed evaluation of how different process parameter combinations influenced the local microhardness distribution. Because the same stacking sequence was maintained throughout the second experimental stage, the resulting profiles can be directly compared for tool rotational speed and welding speed. No microhardness measurements were taken in areas with joint defects.

The microhardness of the upper layer (AA7075) is about 21% lower than that of the AA7075 base material (Figure 23). This reduction indicates that FSW-induced thermal and mechanical conditions affect AA7075. Variations observed approximately 5–6 mm from the joint center also indicate changes in microhardness outside the central stirred region.

The microhardness of the middle layer (AA2024) is about 16% lower than that of the AA2024 base material (Figure 23). As with AA7075, this decrease can be attributed to the thermal and mechanical effects of the FSW process and local material redistribution within the joint.

The microhardness of the lower AA6061-O layer increased from approximately 41 HV in the base material to approximately 49 HV after FSW, a rise of about 19%. This was the only one of the three layers that exhibited an increase in average microhardness after FSW (Figure 23). Unlike AA7075-T6 and AA2024-T3, AA6061 was used in the O temper, which has a relatively low initial microhardness. The observed increase may be associated with severe plastic deformation and local microstructural changes in the FSW-affected regions. However, in the absence of quantitative microstructural and precipitate characterization, the mechanisms responsible for this increase cannot be directly established from the results of the present study.

Overall, the microhardness results demonstrate distinct responses among the three aluminum alloys to the FSW process. Compared with the corresponding base materials, the average microhardness decreased by approximately 21% for AA7075 and 16% for AA2024, whereas it increased by approximately 19% for AA6061. These differences highlight the influence of the initial material condition and the local thermal–mechanical and material-flow conditions developed within the multilayer joint.

## 4. Discussion

The study of FSW joining technology for the three aluminum alloys shows that joint characteristics vary with both the relative position of the alloys during the process and the process parameters used. First-stage experiments showed that the aluminum alloy placed on top, in direct contact with the tool shoulder, influences the process’s thermomechanical behavior and affects material flow and defect formation. This effect can be attributed to the direct interaction between the upper layer and the tool shoulder, which plays an important role in heat generation and material plasticization during FSW. Consequently, changing the upper-layer alloy alters the thermal response of the multilayer structure and the conditions governing material flow through the joint thickness. In the initial experimental plan, Experiment 3, with the AA7075–AA2024–AA6061 stacking sequence, exhibited the highest measured surface temperature and the fewest observed defects among the three configurations.

Macrostructural and microstructural analysis of first-stage FSW joints confirmed the importance of aluminum alloy positioning within the joint stack. In the first-stage experiments, material flow differed in the Stir Zone and the Thermo-Mechanically Affected Zone between the tool’s advancing and retreating sides. The boundaries between the alloys remained visible in certain regions of the joint, indicating that the investigated processing conditions did not achieve complete homogenization.

The relevance of the internal defects observed in the present study is further supported by the authors’ previous investigation [27], which combined mechanical testing with X-ray microtomography and acoustic emission to show that defect development and crack concentration influenced the mechanical response of a three-layer FSW joint.

For the six second-stage experiments, variations in tool rotational speed and welding speed affected the measured surface temperature, axial force, and the resulting FSW joint characteristics. Increasing tool rotational speed raised measured surface temperatures and reduced axial forces, indicating greater thermomechanical softening under the investigated conditions. However, higher surface temperatures did not necessarily improve joint quality. This dependence of joint characteristics on the combination of process parameters is generally consistent with results reported for dissimilar AA2024/AA7075 FSW joints [19], where variations in processing conditions affected weld geometry, defect formation, and mechanical properties. However, the comparison should be made with caution because Ref. [19] investigated a two-alloy joint, whereas the AA7075–AA2024–AA6061 multilayer configuration examined in the present study introduces an additional interface and distinct thermomechanical responses among the three materials. Our results show that increases in measured surface temperature and reductions in axial force associated with higher rotational speed do not necessarily improve macrostructural integrity. This finding indicates that, for dissimilar multilayer configurations, increased thermomechanical input must be considered alongside the stability of material flow and redistribution and cannot be used alone as an indicator of joint quality. The experiment at the minimum tool rotational speed of 650 rpm and the maximum welding speed of 100 mm/min produced the most favorable macrostructure within the investigated parameter range. A similar trend was reported in a previous study on the same AA7075–AA2024–AA6061 stacking sequence [29], where higher rotational speeds produced more defects, while the most favorable macrostructure occurred at lower rotational speeds. These results reinforce the observation that, for this trilayer system, increasing rotational speed does not necessarily improve the joint’s macrostructural characteristics. Taken together, the temperature, axial-force, and macrostructural results suggest a relationship between the process thermomechanical input, material plasticization, and defect formation. Higher tool rotational speed was associated with higher measured surface temperatures and lower axial forces, consistent with increased material plasticization and reduced resistance to tool penetration and material displacement. However, this enhanced thermomechanical response did not systematically result in fewer defects. This behavior indicates that satisfactory joint formation depends on a balance between sufficient material plasticization and controlled flow of the three alloys around the tool, rather than solely on maximizing thermal input. The persistence of boundaries between the alloys and the localization of defects at specific interfaces further support the role of local material distribution and flow in multilayer joint formation.

The results therefore indicate a process window rather than a simple relationship between increasing temperature and improved joint quality. Sufficient thermal softening is required for material flow, but greater thermomechanical input does not necessarily consolidate the three layers effectively.

This is especially evident when comparing Experiments 4.2 and 4.3. Experiment 4.2 (1100 rpm and 60 mm/min) exhibited substantially more defects, whereas Experiment 4.3 (650 rpm and 100 mm/min) produced the most favorable macrostructure. The zone-based analysis further shows that defect formation was not spatially random. Defects preferentially developed in specific layers and on specific sides of the joint. This suggests that local material-flow conditions and interfacial interactions are important factors in consolidating the multilayer structure.

The quantitative defect assessment further shows that joint integrity cannot be adequately characterized by defect occurrence alone. Across the six second-stage conditions, the defect area fraction ranged from 0.138% to 4.735%, while the maximum defect dimension varied from 0.264 to 2.268 mm. A particularly relevant comparison is between Experiments 4.2 and 4.3, where the defect area fraction decreased from 4.735% to 0.138% and the maximum defect dimension decreased from 1.930 to 0.264 mm. Thus, the processing condition corresponding to Experiment 4.3 reduced both the overall defect content and the size of the dominant discontinuities. Conversely, Experiment 4.6 shows that a lower overall defect fraction does not necessarily imply lower defect severity, since this condition exhibited the largest individual defect despite having a lower defect area fraction than Experiments 4.2 and 4.4. These results support using defect population, area fraction, individual defect size, and spatial distribution when assessing the macrostructural integrity of multilayer FSW joints.

In Experiment 4.2, defects accumulated predominantly in the upper AA7075 layer, particularly on the advancing side. In contrast, the remaining discontinuities in Experiment 4.3 were confined to the advancing side within the AA2024/AA6061 region. This location aligns with microstructural observations, which revealed a localized defect on the advancing side near the AA2024/AA6061 interface. The agreement between the quantitative zone-based defect analysis and the microstructural observations provides independent evidence that the residual discontinuities under the 650 rpm–100 mm/min condition were preferentially associated with the AA2024/AA6061 region on the advancing side. Although the two analyses were performed at different observation scales and therefore should not be interpreted as measurements of the same individual defect, their consistent spatial localization strengthens the identification of this interfacial region as a preferential location for residual discontinuity formation under this processing condition.

The presence of three dissimilar alloys makes material flow more complex than in conventional single-alloy FSW. Each layer responds differently to the thermal and deformation cycle. Therefore, local flow compatibility between adjacent layers is important to prevent defects. The persistence of visible interfaces after welding supports this interpretation. Although substantial material redistribution occurred, the three alloys did not fully homogenize.

The microhardness distribution highlights the distinct responses of the three alloys to the thermal cycles and plastic deformation generated during FSW, and this behavior should also be interpreted in relation to each material’s initial metallurgical condition. Compared with the corresponding base materials, AA7075-T6 and AA2024-T3 exhibited average microhardness reductions of approximately 21% and 16%, respectively, whereas AA6061-O showed an increase of approximately 19%. For AA7075-T6 and AA2024-T3, which initially exhibit relatively high hardness levels associated with their respective temper conditions, thermomechanical exposure during FSW may alter the precipitation state and local microstructure, contributing to hardness reduction in the process-affected regions. In contrast, AA6061 in the O temper has substantially lower initial hardness. In this case, severe plastic deformation, microstructural refinement, and local material redistribution in the FSW-affected regions may contribute to microhardness values higher than those of the base material. A comparable behavior has been reported for FSW joints involving AA6061 in the annealed condition, where microhardness values higher than those of the base material were observed after processing [12]. This similar trend supports the interpretation that the initial metallurgical condition plays an important role in the local hardness response to the FSW thermomechanical cycle. Thus, the contrasting responses of the three alloys should not be interpreted solely in terms of process temperature, but rather as the result of the interaction among the initial metallurgical condition, the thermomechanical cycle, and local material redistribution. However, without direct precipitate characterization and quantitative microstructural analysis, the proposed metallurgical mechanisms should be regarded as plausible interpretations of the observed microhardness trends rather than mechanisms directly demonstrated in the present study.

Surface roughness analysis of the second-stage experiments showed a decreasing trend with increasing tool rotational speed and decreasing welding speed. At the same time, progressive stabilization along the FSW weld bead was observed. However, the conditions that produced lower surface roughness did not coincide with those that produced the fewest macrostructural defects. Therefore, surface roughness alone cannot be considered a direct indicator of the internal integrity of the FSW joint.

The first- and second-stage results highlight the combined effects of material configuration and FSW parameters on thermomechanical behavior, material flow, and resulting joint characteristics. The AA7075–AA2024–AA6061 configuration, paired with 650 rpm and 100 mm/min, produced the most favorable macrostructural outcome within the investigated parameter range.

Although tensile tests were not repeated in the present experimental campaign, the authors investigated the mechanical behavior of the same stacking sequence and processing conditions used in Experiment 4.3 (AA7075–AA2024–AA6061, 650 rpm and 100 mm/min) in a previous study [31]. Four tensile specimens extracted at successive positions along the weld exhibited maximum tensile forces of 8.71, 16.30, 19.05, and 20.35 kN, respectively. The increase in maximum force along the weld was associated with the progressive stabilization of the FSW process, and the last two specimens showed relatively close values of 19.05 and 20.35 kN [31].

These previously published mechanical results provide complementary context for the quantitative defect assessment in the present study. Experiment 4.3 exhibited the lowest defect area fraction (f_A_ = 0.138%), together with the smallest maximum individual defect area (A_max_ = 0.022 mm^2^) and the maximum defect dimension (L_max_ = 0.264 mm). The previously reported tensile response further shows that specimens extracted after the initial portion of the weld sustained substantially higher maximum loads, reaching 19.05–20.35 kN [31]. These datasets do not establish a direct quantitative relationship between defect content and tensile performance. Nevertheless, both sets of results indicate that the 650 rpm–100 mm/min condition can produce a comparatively sound multilayer joint after process stabilization.

We obtained additional information on the mechanical response of this multilayer system from the previous DIC and acoustic-emission study [32]. These complementary techniques showed that deformation and damage evolution during tensile loading were spatially heterogeneous. For the condition corresponding to Experiment 4.3, the AE analysis identified a preferential localization of damage-related sources within the welded region [32].

Taken together, the present results and the previously published mechanical characterization provide complementary information across different scales. The current analysis quantifies the number, size, and spatial distribution of the remaining defects and characterizes the associated microstructure and local hardness. The earlier tensile, DIC, and AE investigations provide complementary information on load-bearing behavior, strain localization, and damage evolution under the same processing condition [31,32]. This combined evidence supports identifying 650 rpm and 100 mm/min as the most favorable condition within the investigated range from a macrostructural perspective, without implying mechanical optimization.

A limitation of the present study is the limited number of independent experimental replicates. Although we assessed process repeatability at the intermediate condition of 845 rpm and 80 mm/min, we did not replicate the other parameter combinations. Consequently, the differences observed among the experimental conditions reflect process trends within the investigated range and should not be interpreted as statistically significant parameter effects. An extended experimental design with independent replicates for each condition will be required to quantify process variability and to statistically evaluate the individual and combined effects of the FSW parameters.

However, detailed microstructural analysis was limited to the condition selected based on the macrostructural assessment, so the present results do not provide a systematic microstructural comparison across contrasting processing conditions. Furthermore, the microstructural characterization in this study relied on optical microscopy and did not include advanced phase, compositional, or crystallographic analyses such as XRD, SEM/EDS, EBSD, or TEM. Consequently, the present results cannot directly establish phase evolution, local elemental redistribution, crystallographic texture and grain-boundary characteristics, or nanoscale precipitate evolution. These aspects require dedicated advanced characterization and represent an important direction for future investigation. Although the present image-based analysis quantified the two-dimensional defect population in terms of number, total area, area fraction, maximum individual defect area, and maximum defect dimension, it did not provide three-dimensional information on defect morphology and volume. Therefore, complementary three-dimensional characterization, for example, by X-ray micro-computed tomography, would be valuable for assessing the volumetric distribution and connectivity of internal defects.

Another limitation of the present experimental campaign is that we did not systematically conduct global mechanical-performance tests for all six second-stage conditions. Therefore, we cannot establish a direct correlation between quantitative defect characteristics and tensile performance across the investigated parameter range. Previously published tensile, DIC, and AE results for the condition corresponding to Experiment 4.3 provide complementary mechanical context [31,32], but they do not replace a systematic mechanical comparison of all processing conditions. Consequently, based on the observed macrostructural characteristics within the investigated experimental range, 650 rpm and 100 mm/min are the most favorable conditions and should not be interpreted as the optimum in terms of the joint’s mechanical performance.

Future research should therefore extend the present analysis by systematically comparing microstructures across contrasting processing conditions and by replicating mechanical testing, including tensile and fatigue tests, for all investigated parameter combinations. Complementary fractographic analysis and advanced microstructural characterization would enable direct relationships among process parameters, quantitative defect characteristics and their spatial distribution, microstructure, local hardness, and global mechanical performance. This approach would also enable assessment of the mechanical significance of the defect distributions identified in the present study and more rigorously define the processing window for multilayer AA2024/AA6061/AA7075 FSW joints.

## 5. Conclusions

This study examined how material configuration and key FSW process parameters affect the fabrication of a multi-layer joint from aluminum alloys AA2024, AA6061, and AA7075. Based on the experimental results, the following conclusions can be drawn:

- The relative positioning of aluminum alloys in the welded stack affects thermomechanical behavior, material flow, and defect formation. Among the first-stage experiments analyzed, Experiment 3, with the AA7075–AA2024–AA6061 configuration, had the fewest observed defects and was therefore selected for the second-stage experimental plan.
- For the second-stage experimental plan, the process parameters affected the measured surface temperature and the axial force. Increasing the tool rotational speed increased the measured surface temperature and reduced the axial force. However, higher surface temperatures did not necessarily correspond to improved joint quality.
- The quantitative defect assessment showed a strong dependence of macrostructural integrity on the investigated process parameters. The defect area fraction ranged from 0.138% to 4.735%, and the maximum defect dimension ranged from 0.264 to 2.268 mm, indicating that both overall defect content and individual defect severity should be considered when evaluating multilayer joint integrity. Experiment 4.3 at 650 rpm and 100 mm/min yielded the most favorable macrostructural outcome, with the lowest defect area fraction (0.138%), the smallest maximum individual defect area (0.022 mm^2^), and the smallest maximum defect dimension (0.264 mm). The remaining discontinuities were localized on the advancing side in the AA2024/AA6061 region rather than distributed throughout the multilayer cross-section. Microstructural analysis revealed well-developed material flow, although visible boundaries between the alloys indicated incomplete homogenization. Previously published tensile results for the same stacking sequence and nominal processing parameters [31] provide complementary mechanical context, with maximum tensile forces reaching 19.05–20.35 kN after process stabilization. However, because we did not systematically evaluate mechanical performance across all investigated conditions, this parameter combination should not be considered mechanically optimized.
- The three alloys exhibited distinct microhardness responses after FSW. Compared with the corresponding base materials, average microhardness decreased by approximately 21% for AA7075 and 16% for AA2024, whereas AA6061 increased by approximately 19%. These differences reflect each alloy’s distinct response to the thermal–mechanical conditions and local material redistribution during FSW.

Overall, the results show that the stacking sequence and process parameters jointly govern the thermomechanical response, material flow, defect formation and localization, and local properties of multilayer FSW joints. Importantly, higher measured temperatures or lower axial forces did not necessarily improve macrostructural integrity, underscoring the need to assess FSW joint quality using correlated thermomechanical, defect, microstructural, and local mechanical indicators rather than a single process response.

## Figures and Tables

**Figure 1:**
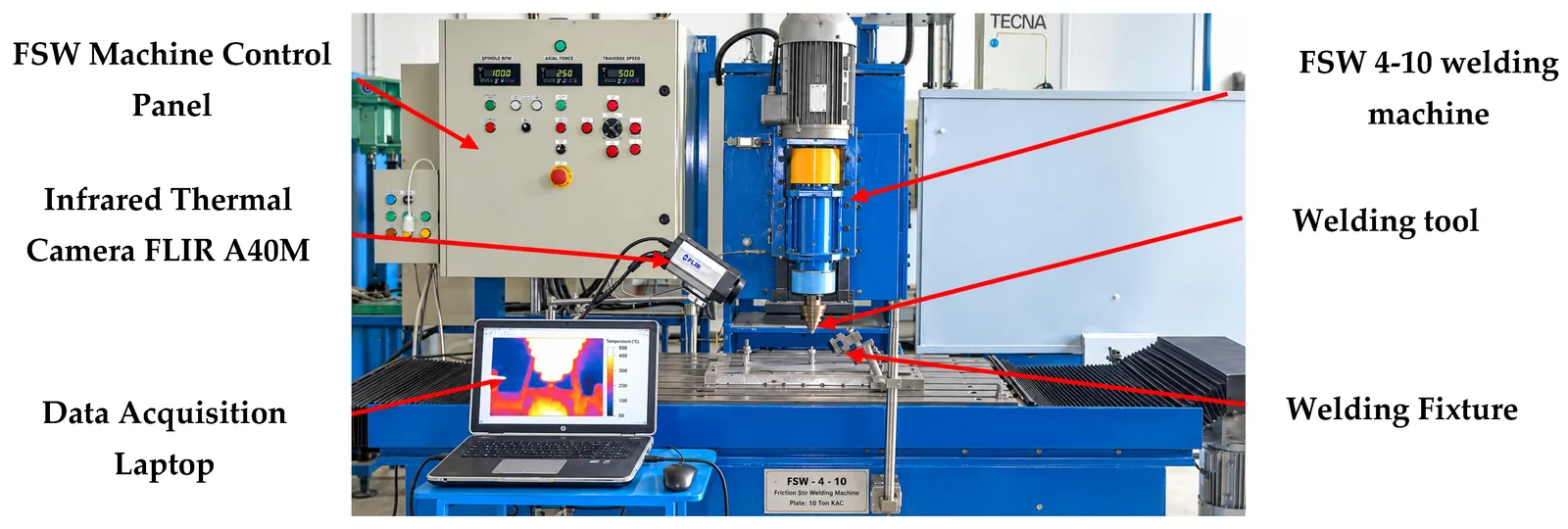
Experimental setup used for the FSW process.

**Figure 2:**
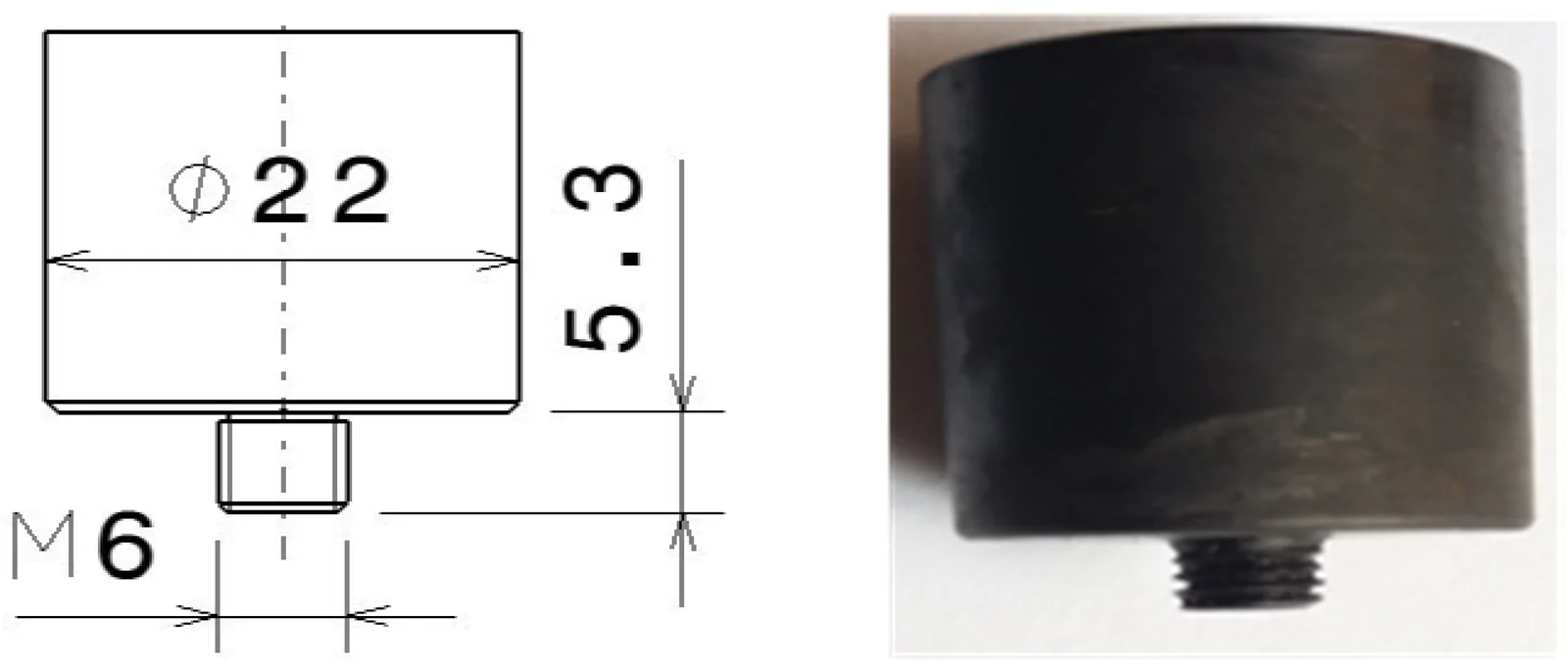
Geometry, main dimensions, and photograph of the FSW tool used in the experiments.

**Figure 3:**
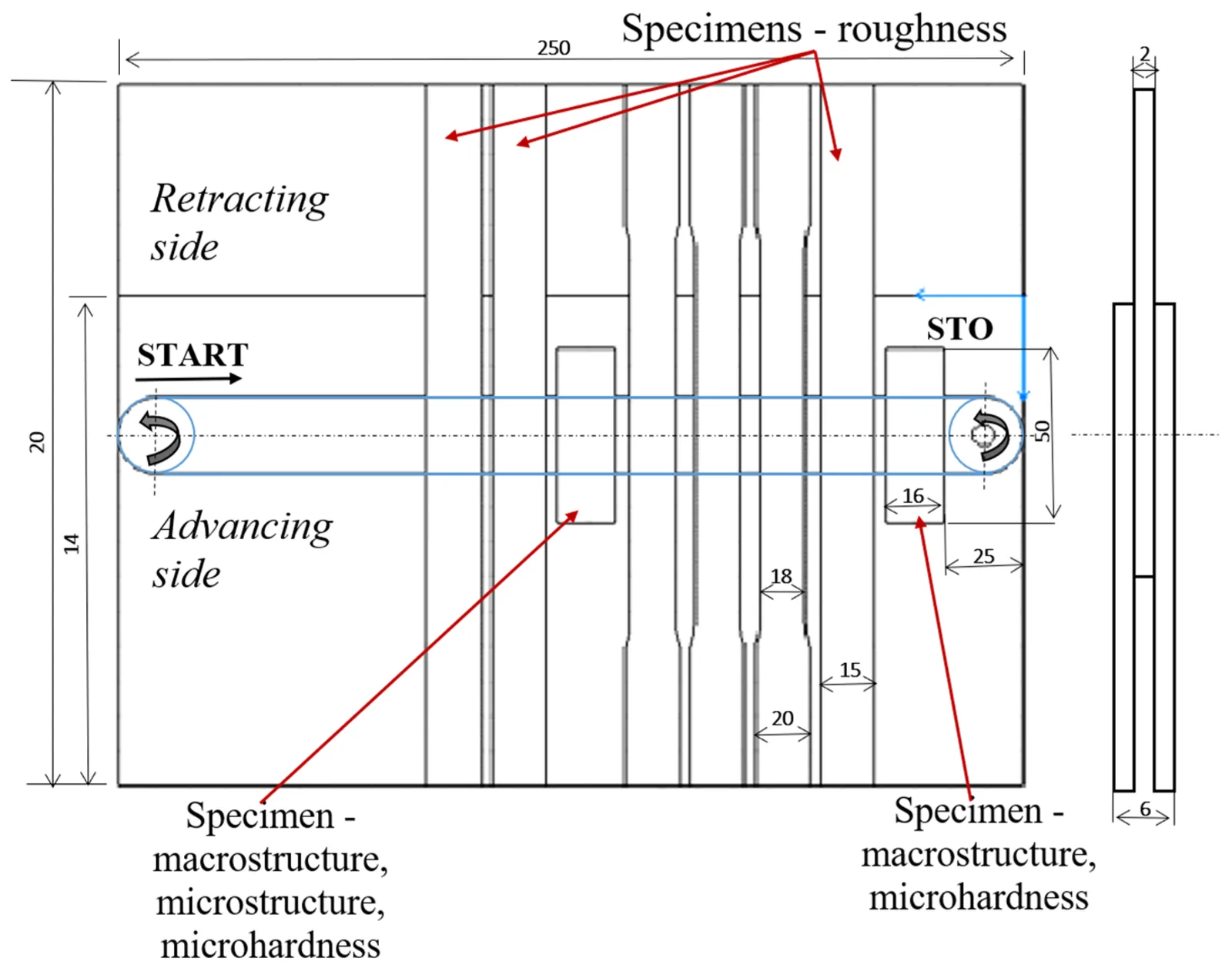
Characteristics of the sample defined within the first-stage welded structures.

**Figure 4:**
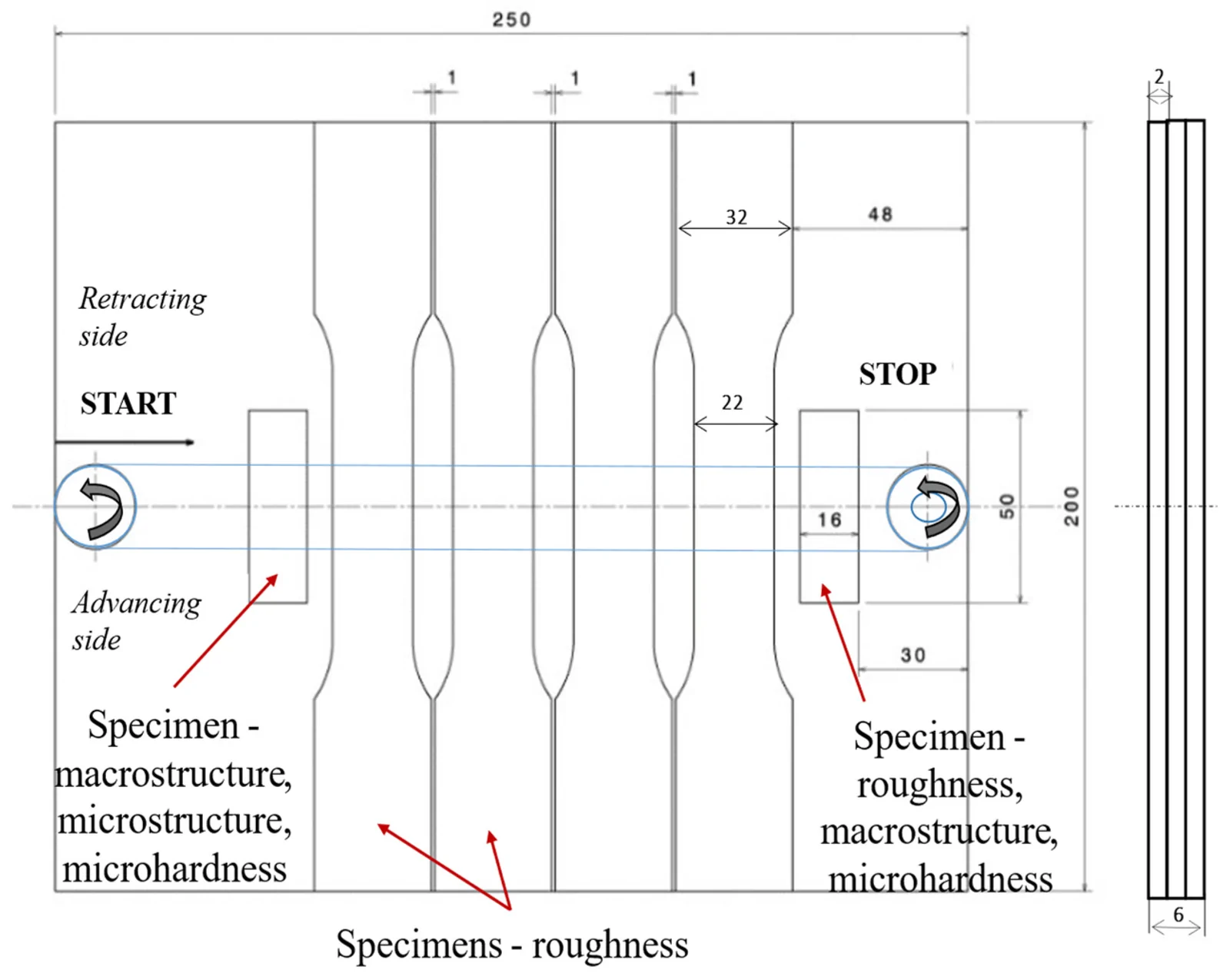
Characteristics of the sample defined within the second-stage welded structures.

**Figure 5:**
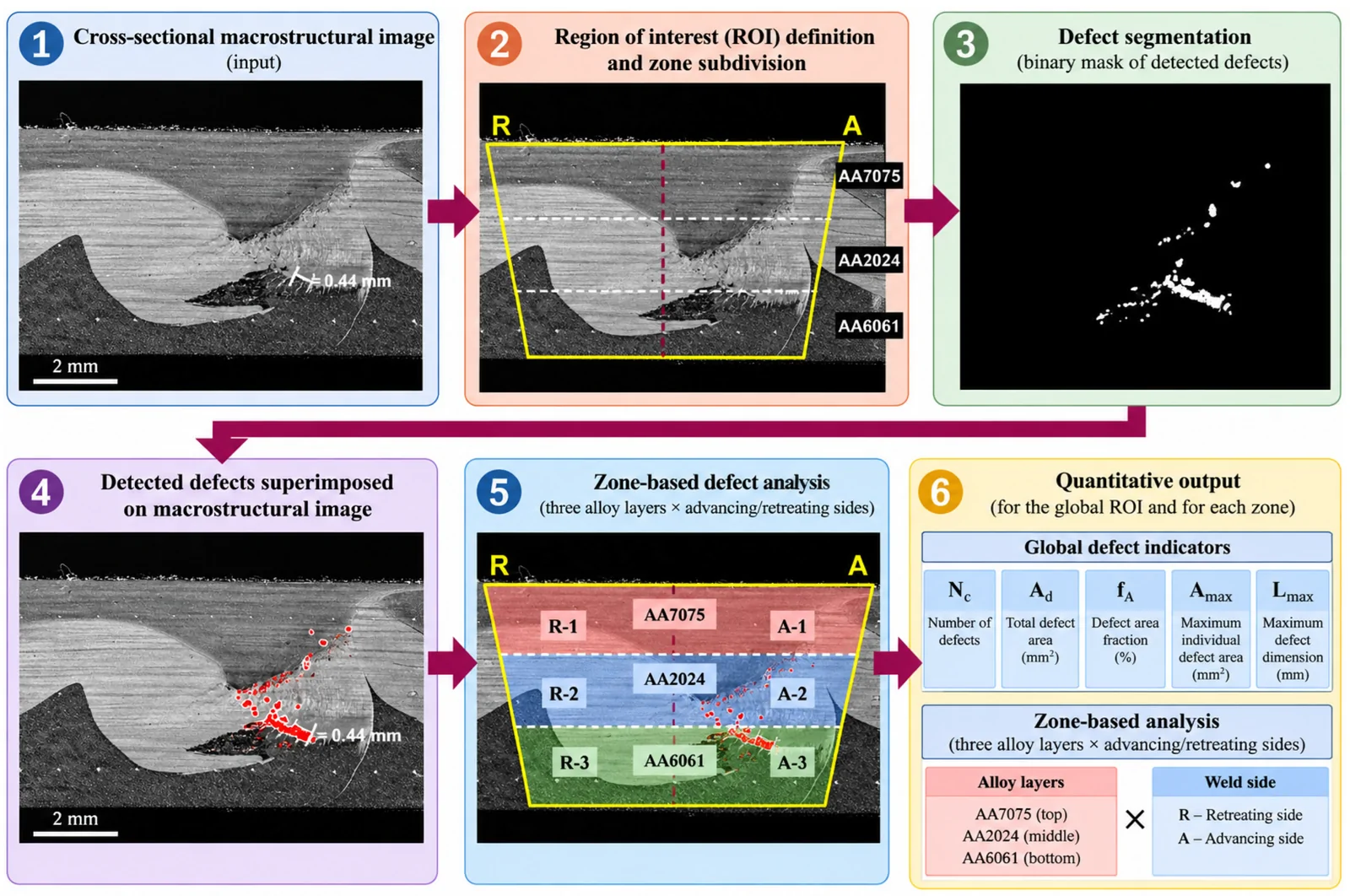
Workflow for the quantitative assessment of macrostructural defects in multilayer FSW joints.

**Figure 6:**
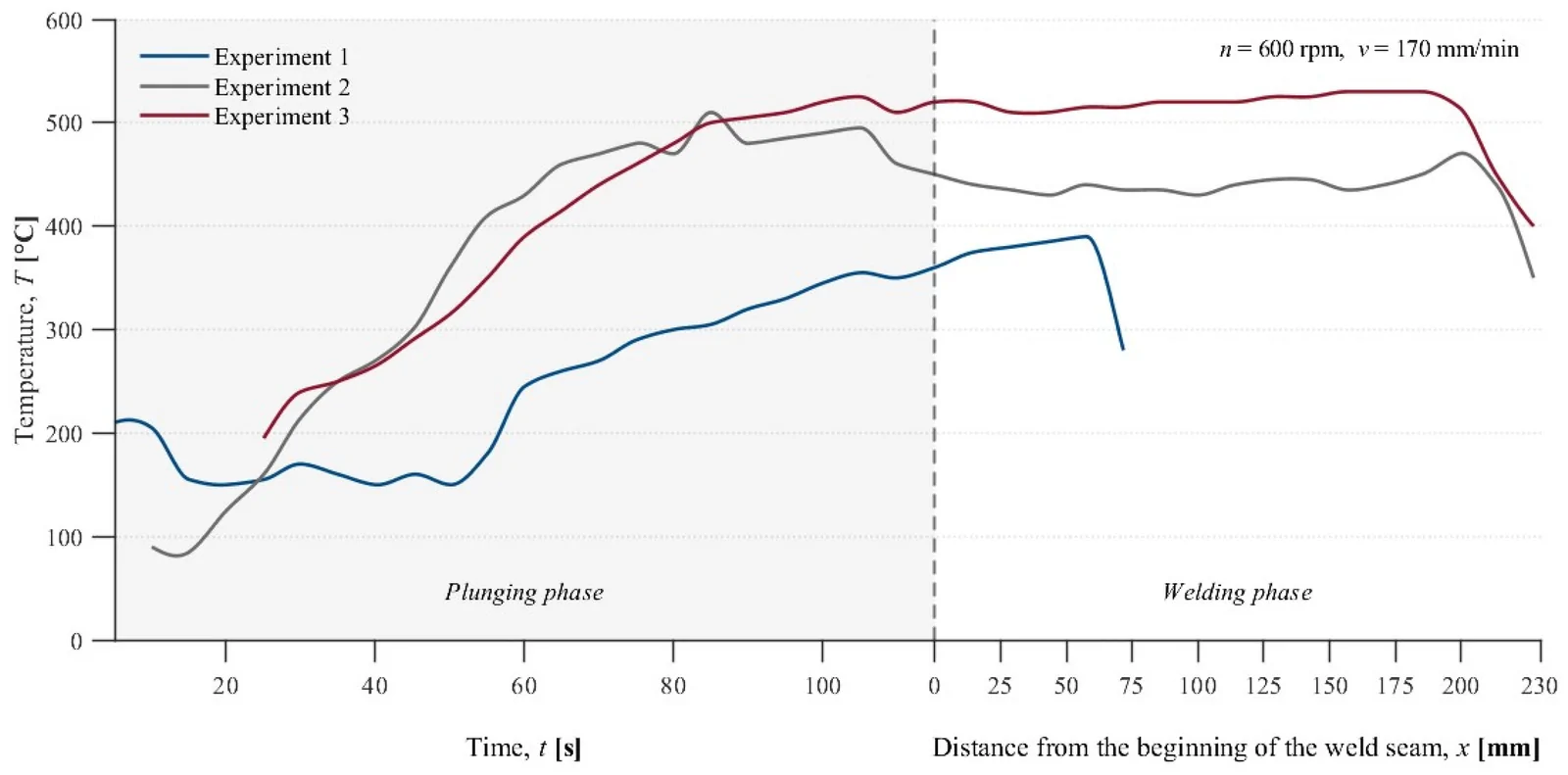
Surface temperature evolution during the first-stage FSW experiments.

**Figure 7:**
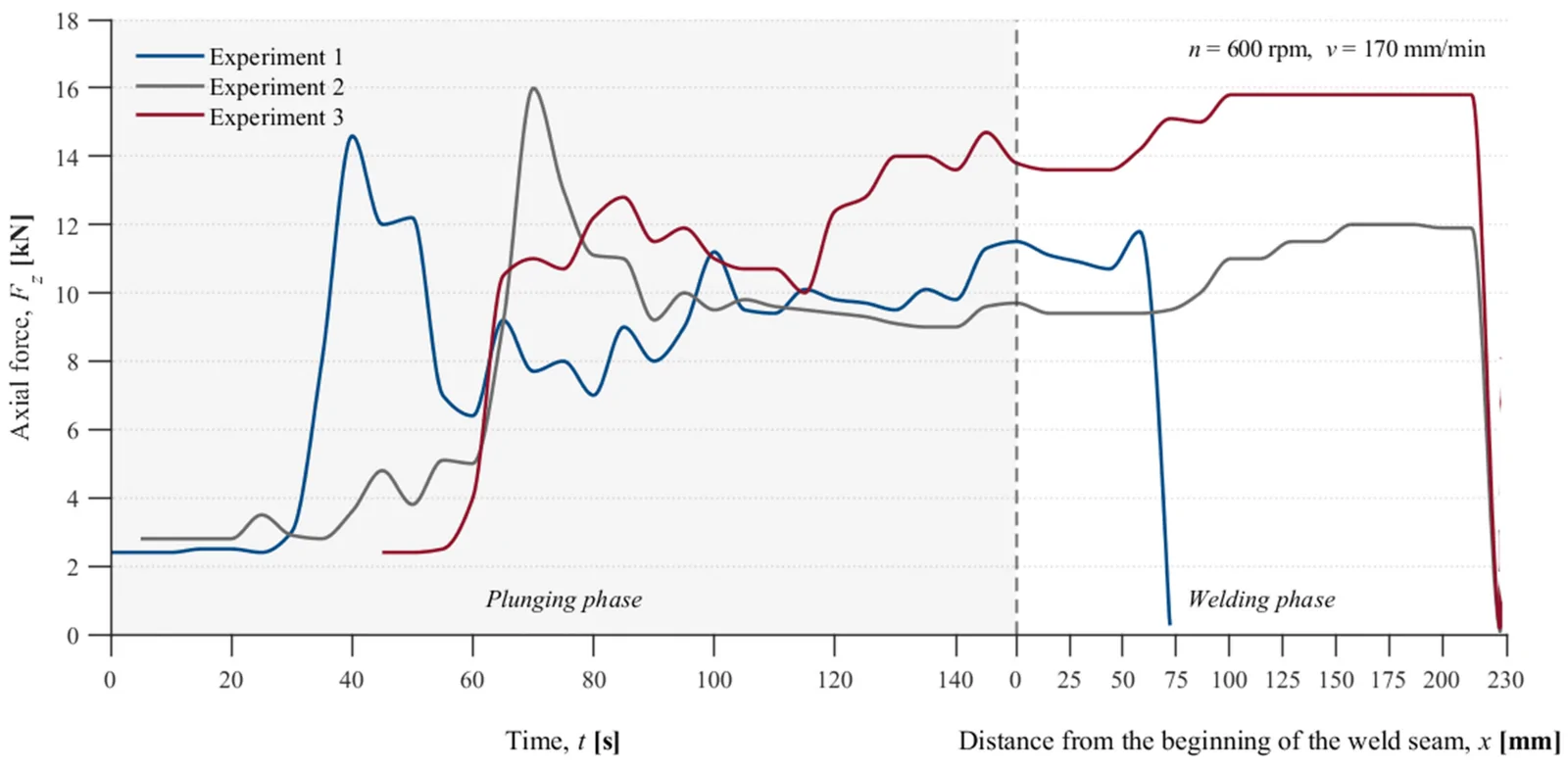
Axial Force evolution during the first-stage FSW experiments.

**Figure 8:**
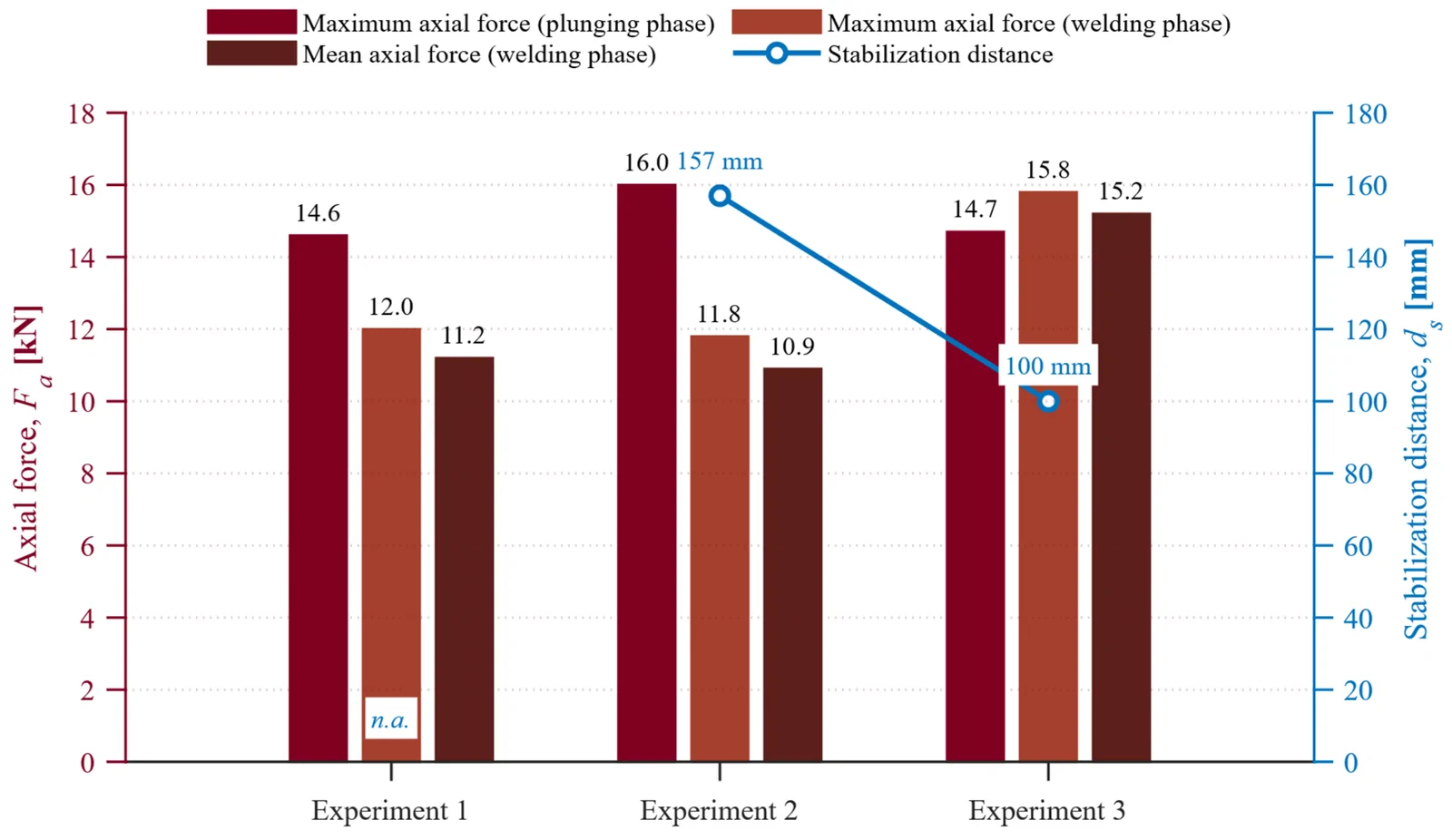
The graphical representation of the values of the specific quantities associated with the axial force for the first-stage FSW experiments. “n.a.” indicates “not applicable”.

**Figure 9:**
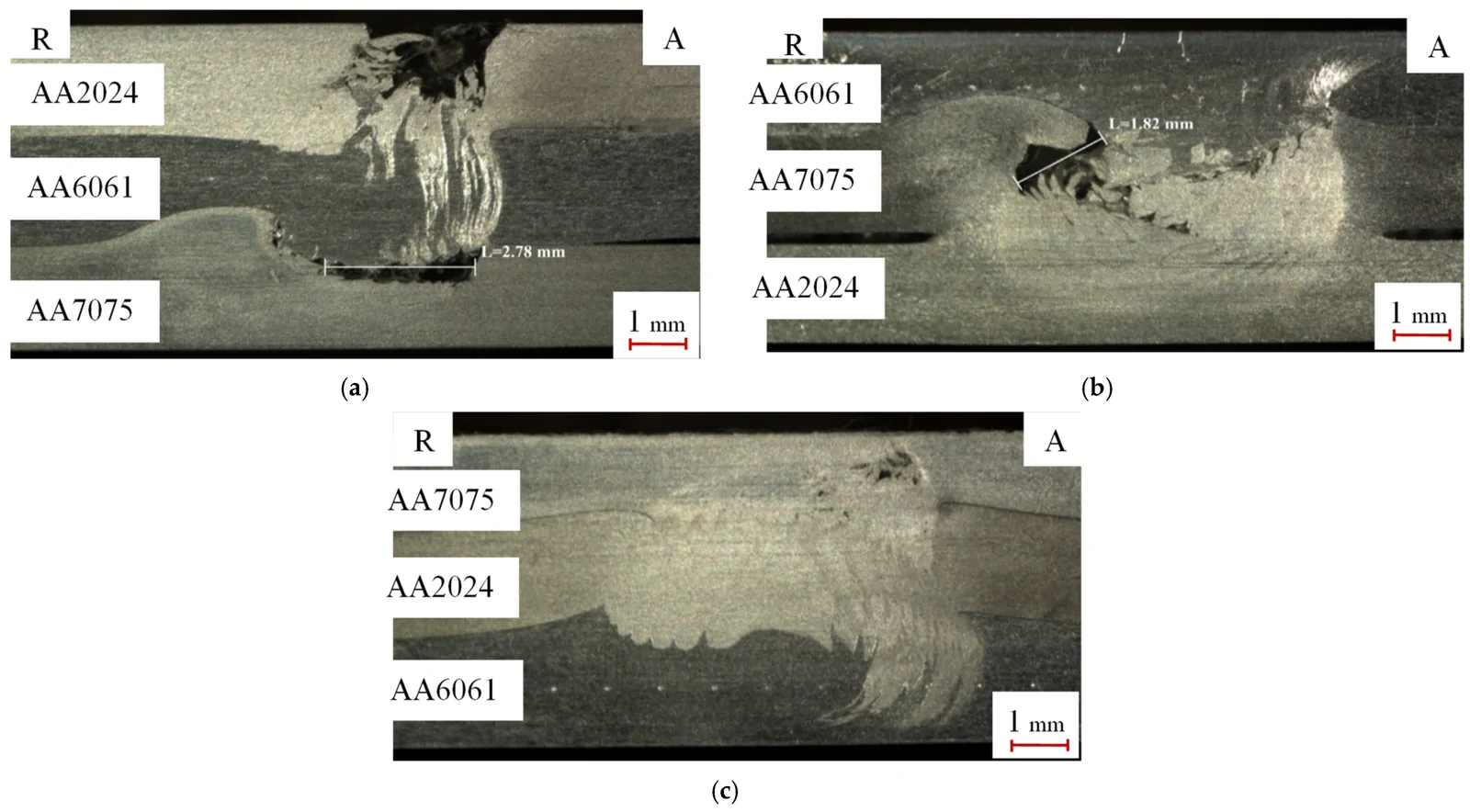
Macrostructure of welded structures at 140 mm from the weld bead’s starting point: (**a**) Experiment 1; (**b**) Experiment 2; (**c**) Experiment 3.

**Figure 10:**
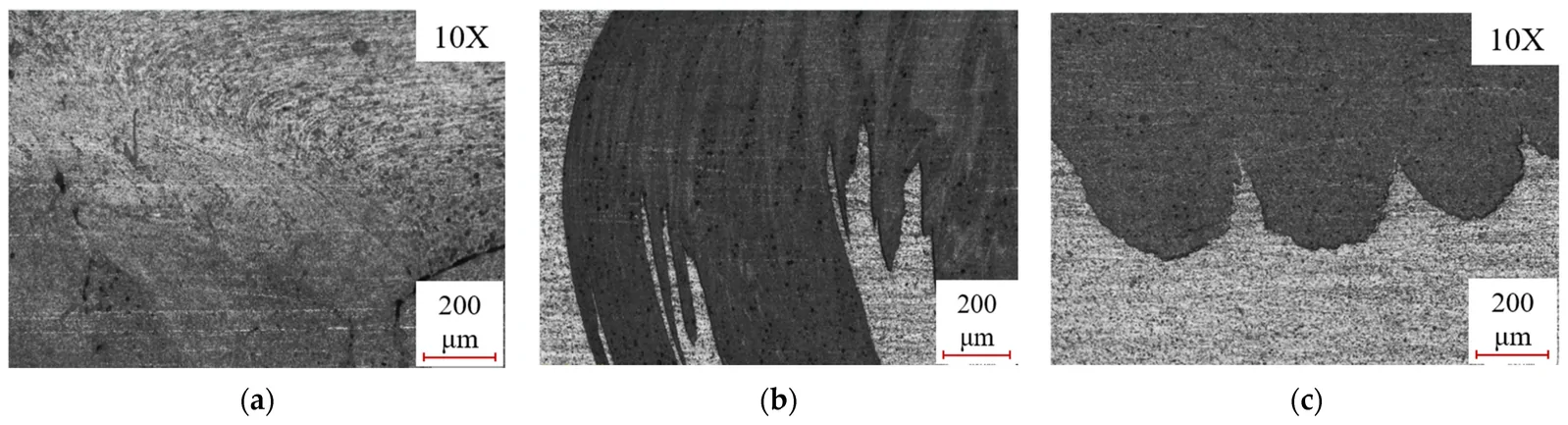
Microstructure of welded structures at 140 mm from the weld bead’s starting point: (**a**) microstructure in the SZ on the retreating side at the AA7075/AA2024 interface; (**b**) microstructure in the TMAZ on the advancing side at the AA2024/AA6061 interface; (**c**) microstructure in the SZ on the retreating side at the AA2024/AA6061 interface.

**Figure 11:**
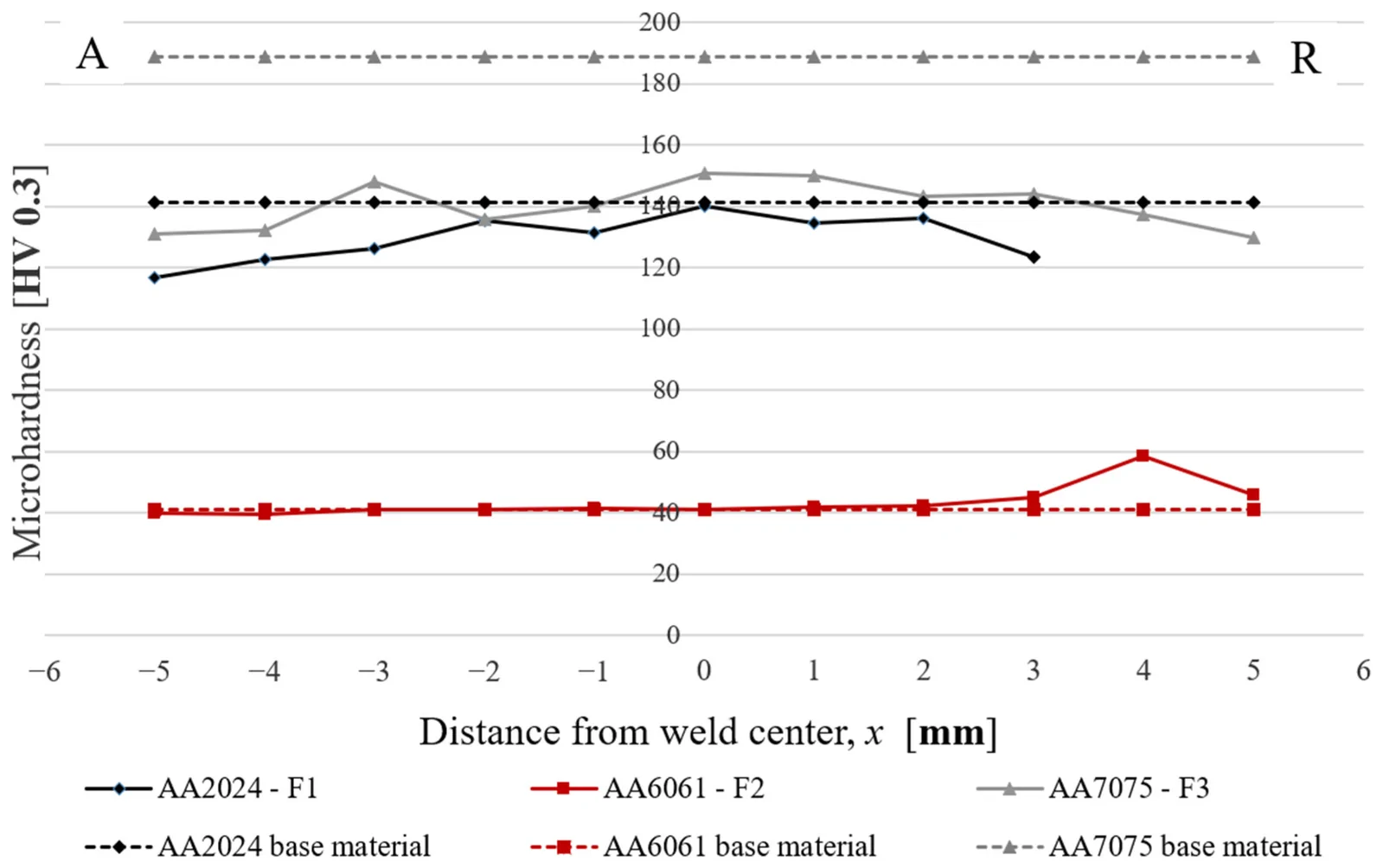
The microhardness variation for Experiment 1 (AA2024–AA6061–AA7075).

**Figure 12:**
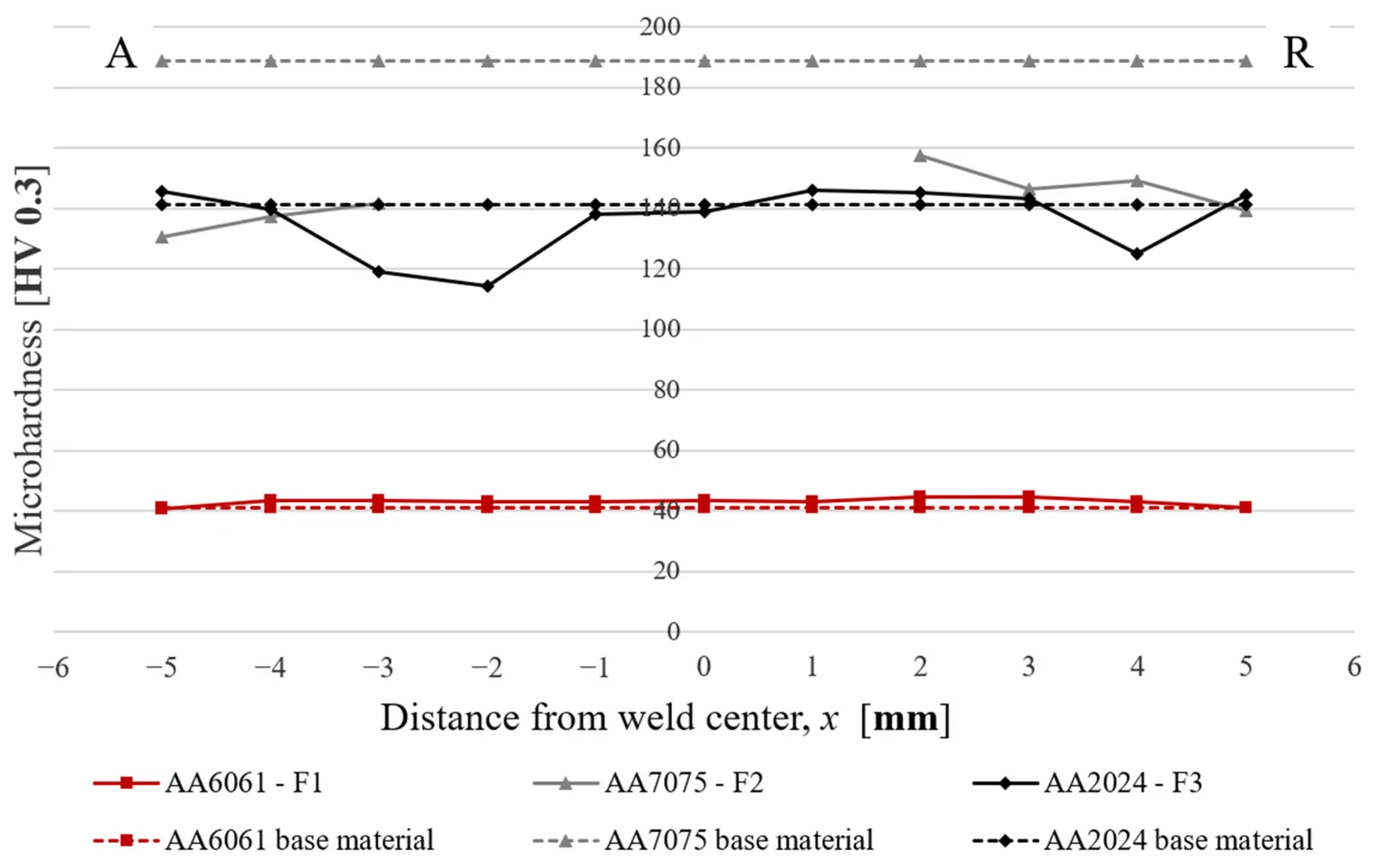
The microhardness variation for Experiment 2 (AA6061–AA7075**–**AA2024).

**Figure 13:**
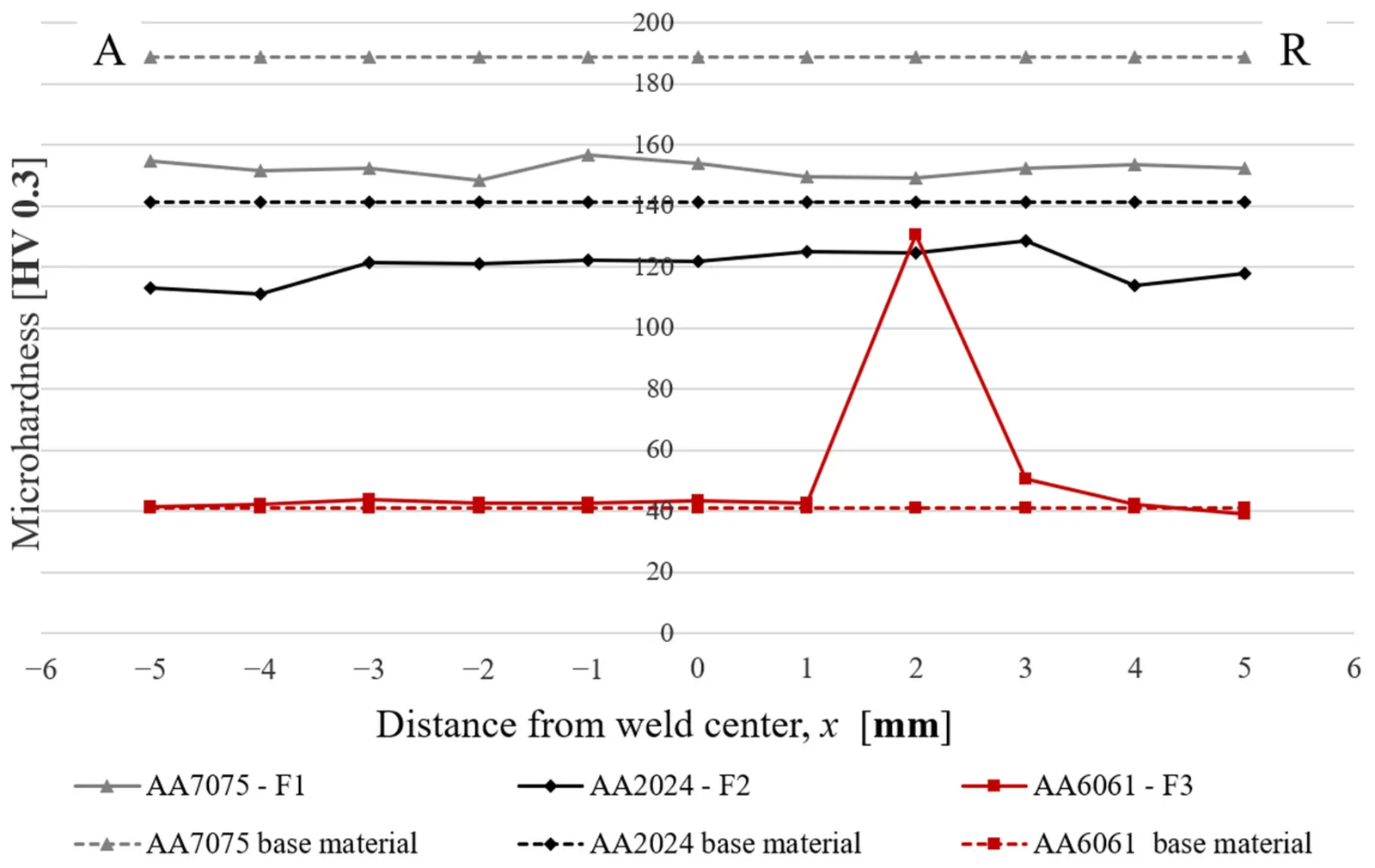
The microhardness variation for Experiment 3 (AA7075**–**AA2024**–**AA6061).

**Figure 14:**
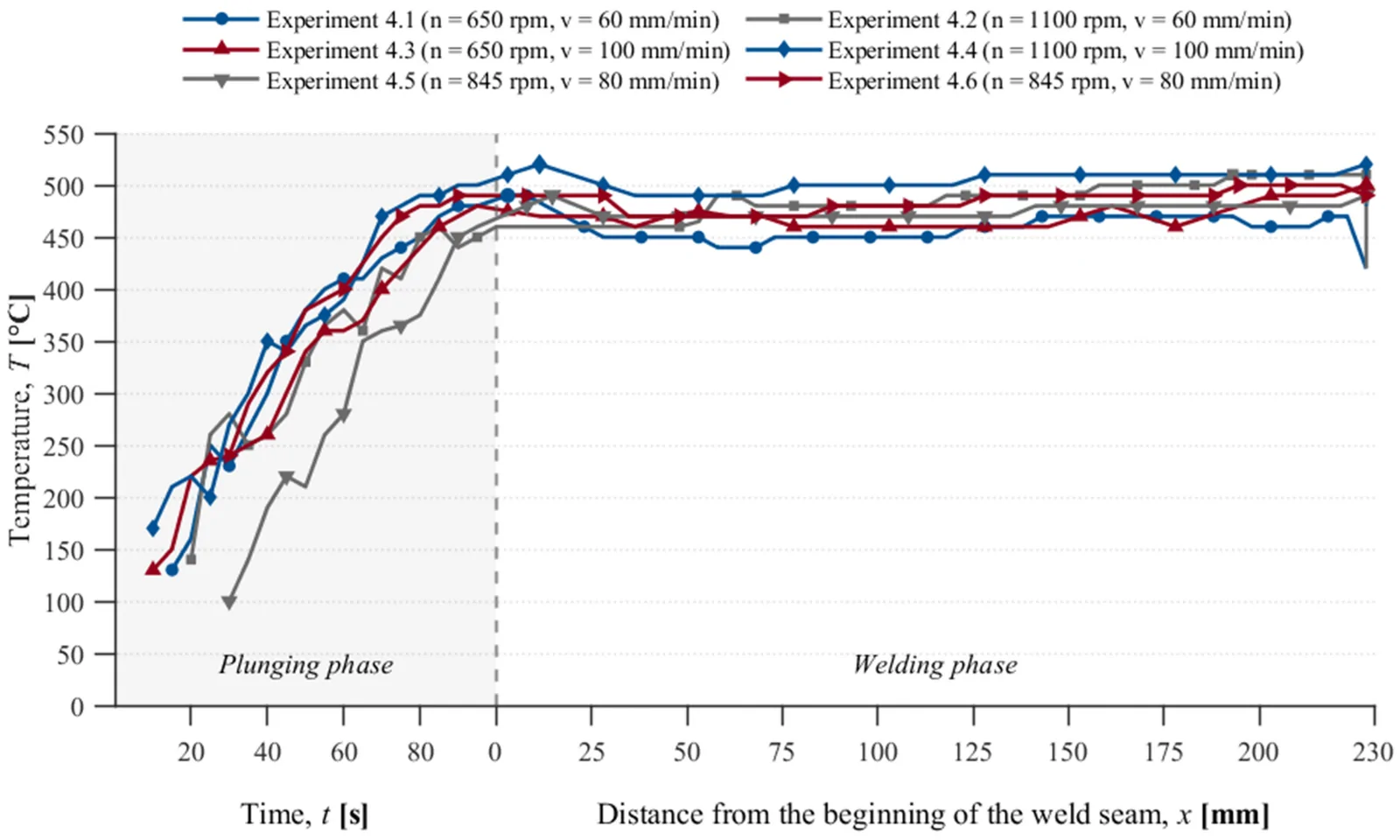
Surface temperature evolution during the second-stage FSW experiments.

**Figure 15:**
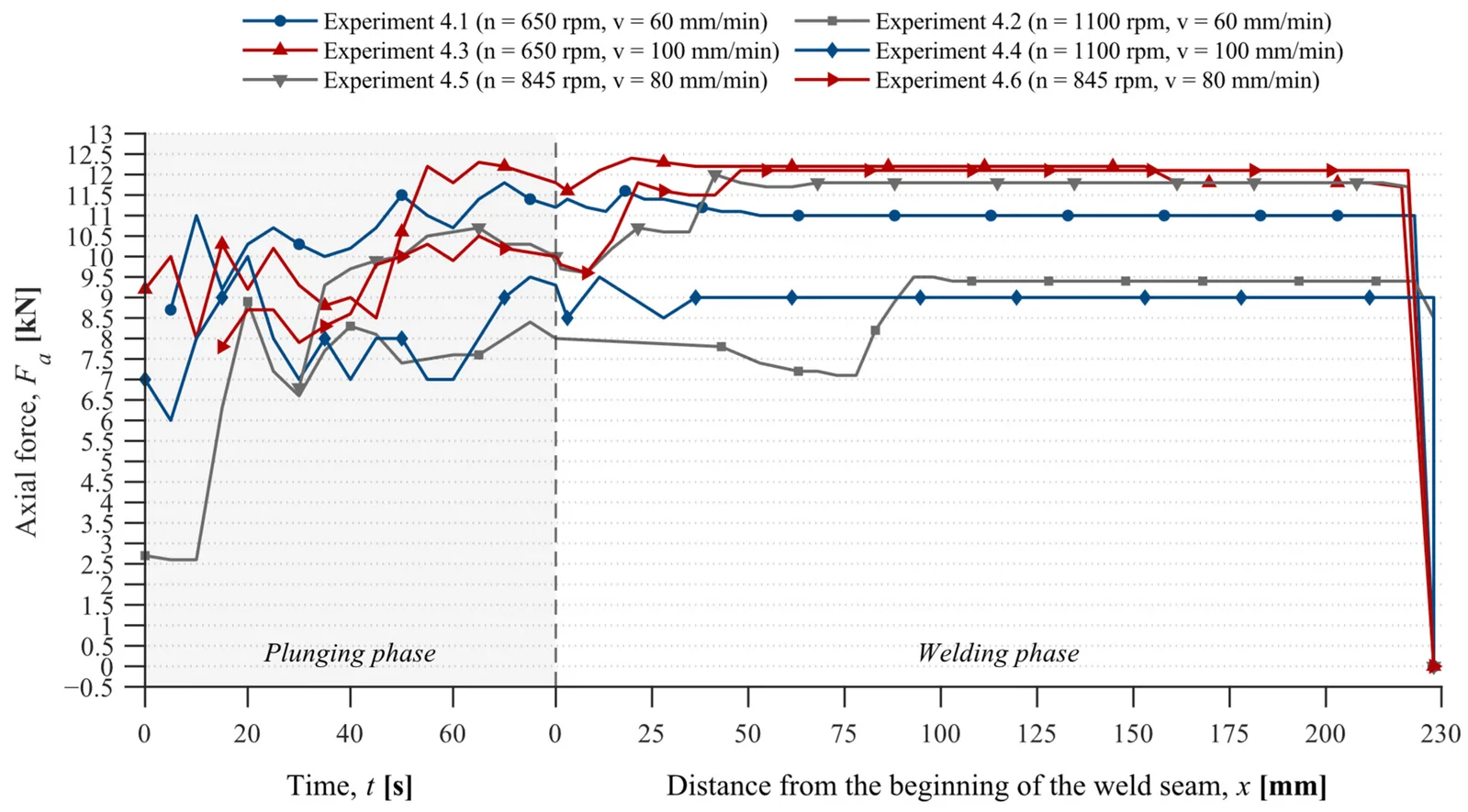
Axial Force evolution during the second-stage FSW experiments.

**Figure 16:**
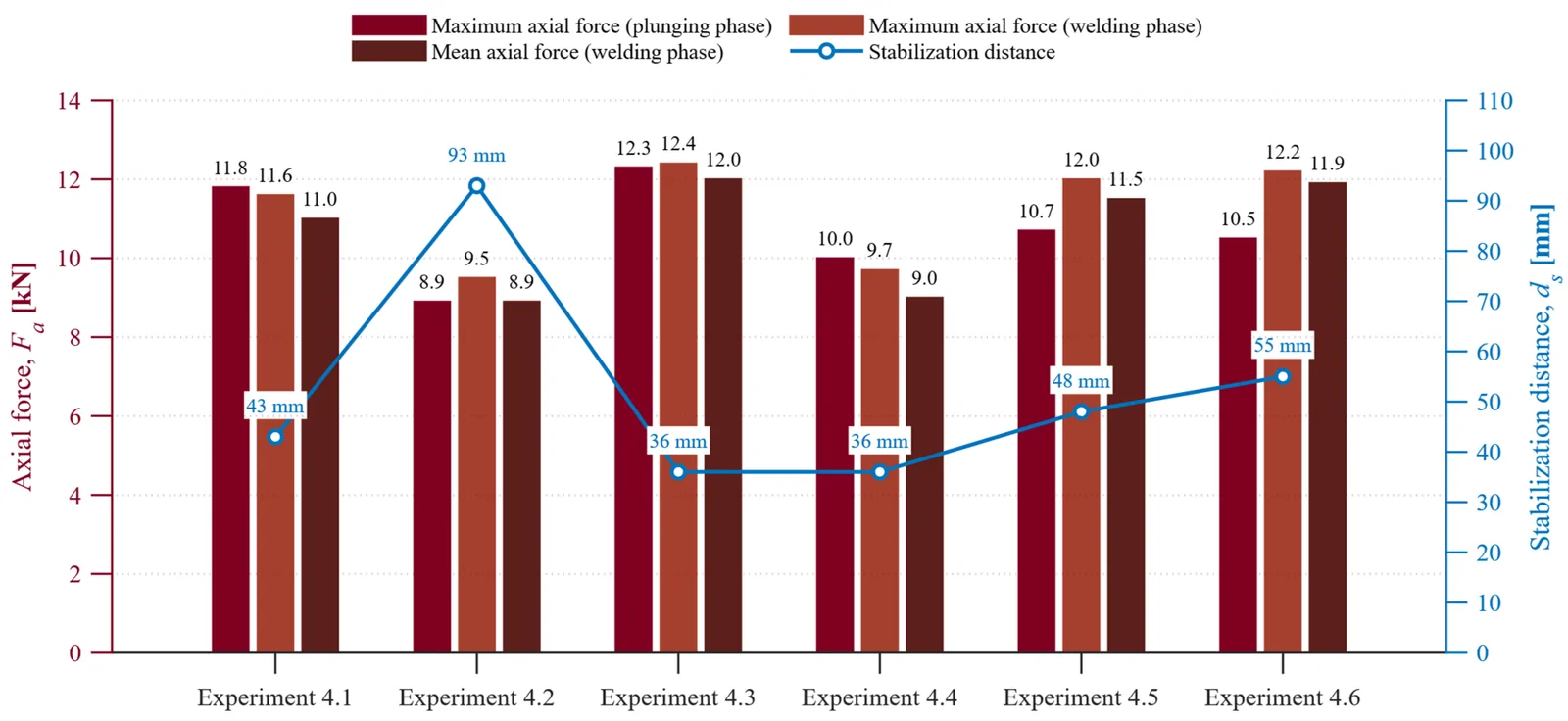
The graphical representation of the values of the specific quantities associated with the axial force for the second-stage FSW experiments.

**Figure 17:**
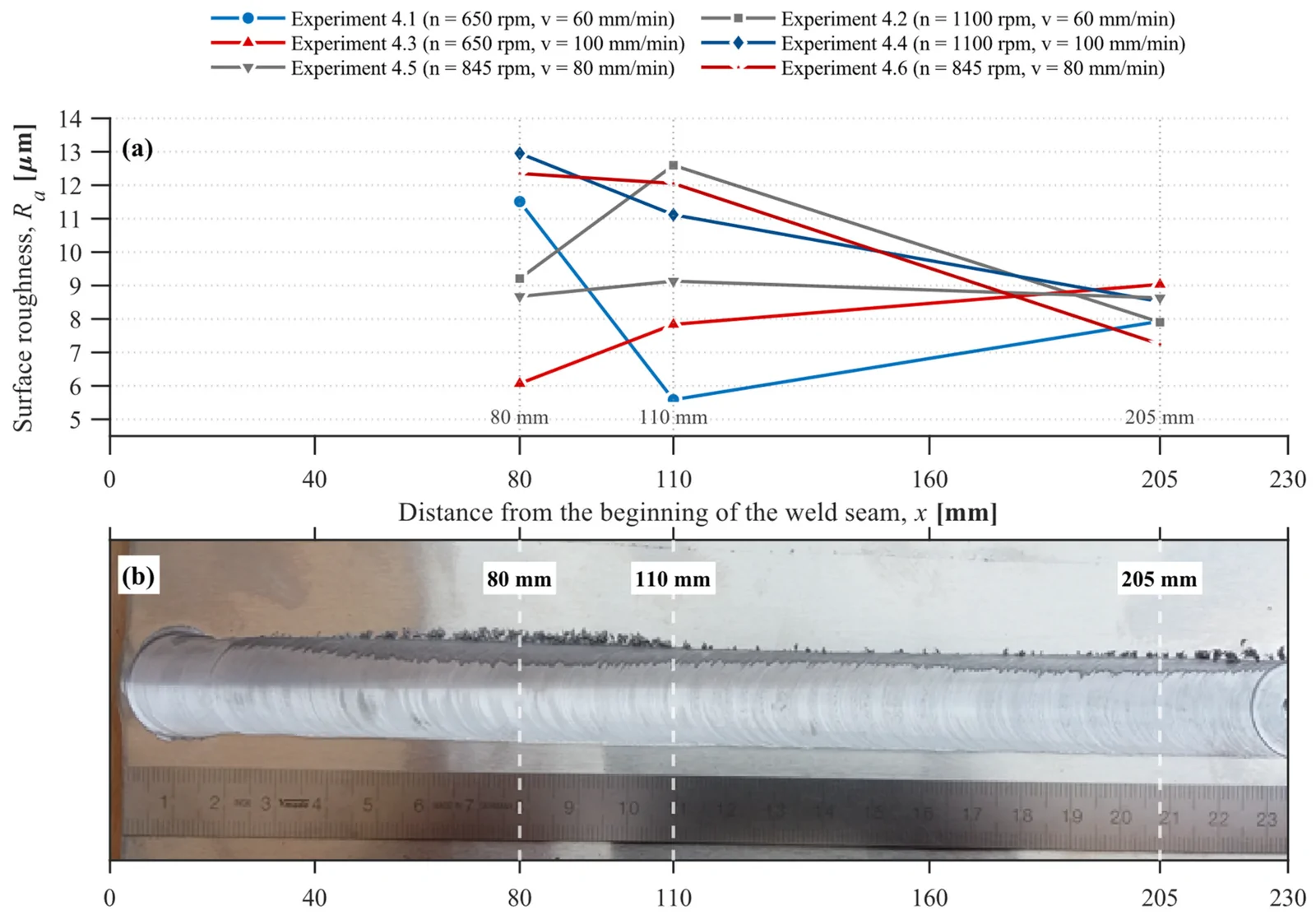
Average roughness values in relation to their location along the weld bead: (**a**) variation of the average surface roughness for the six experimental conditions at the selected measurement positions; (**b**) weld bead image indicating the corresponding measurement locations at 80, 110, and 205 mm from the beginning of the weld seam.

**Figure 18:**
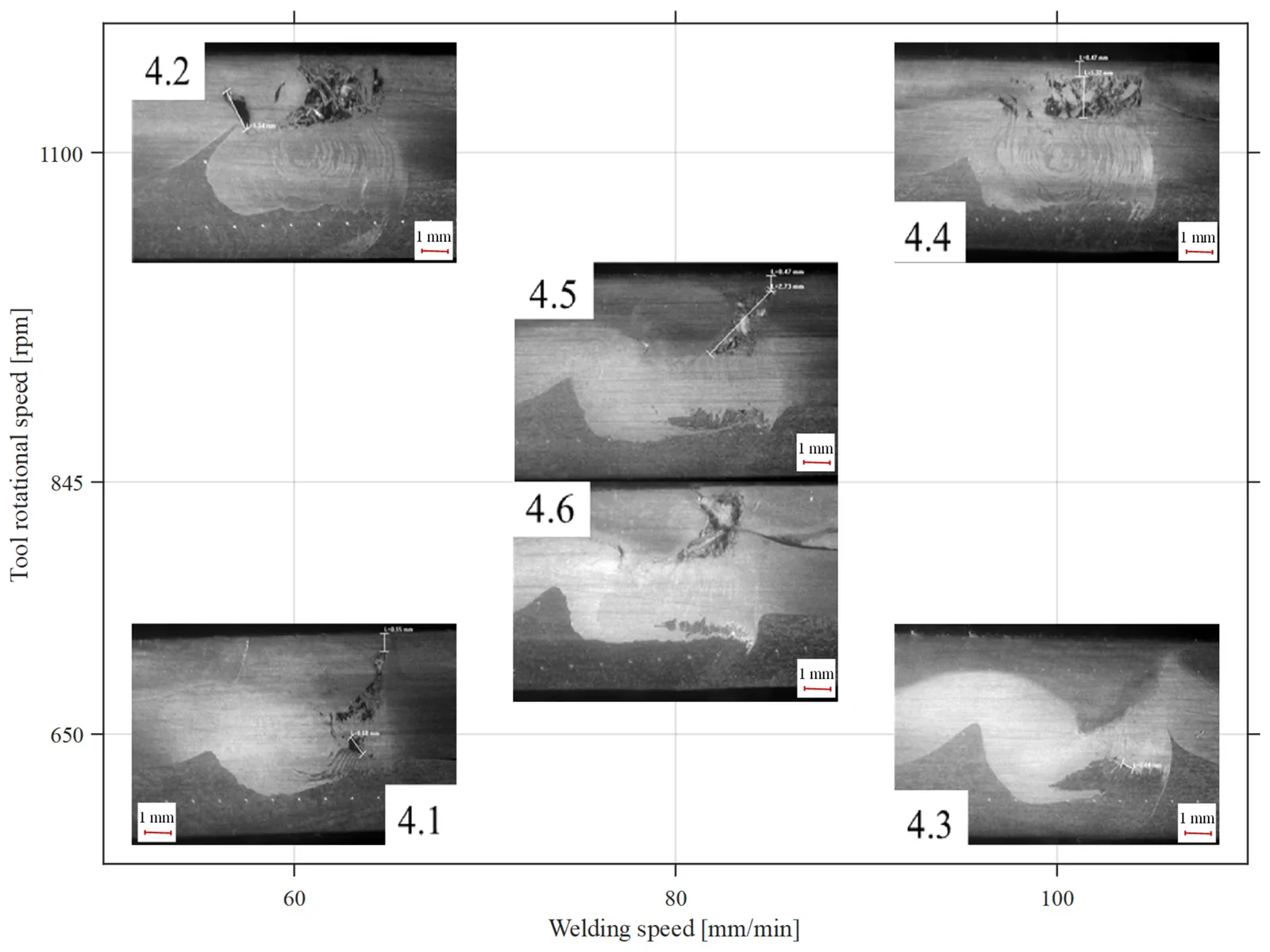
Macrostructures of the FSW joints obtained under different combinations of tool rotational speed and welding speed.

**Figure 19:**
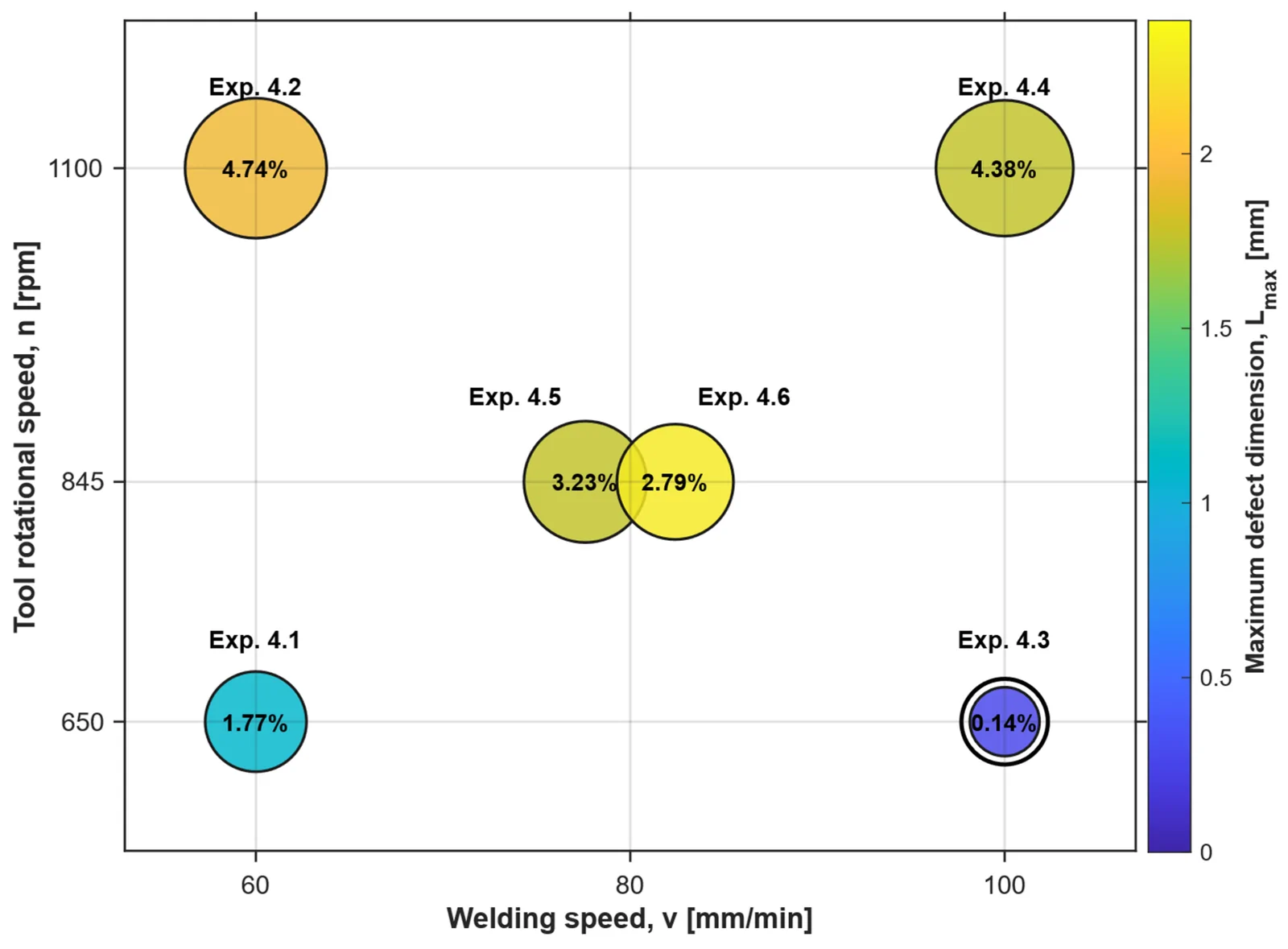
Quantitative comparison of defect characteristics for the six second-stage experimental conditions.

**Figure 20:**
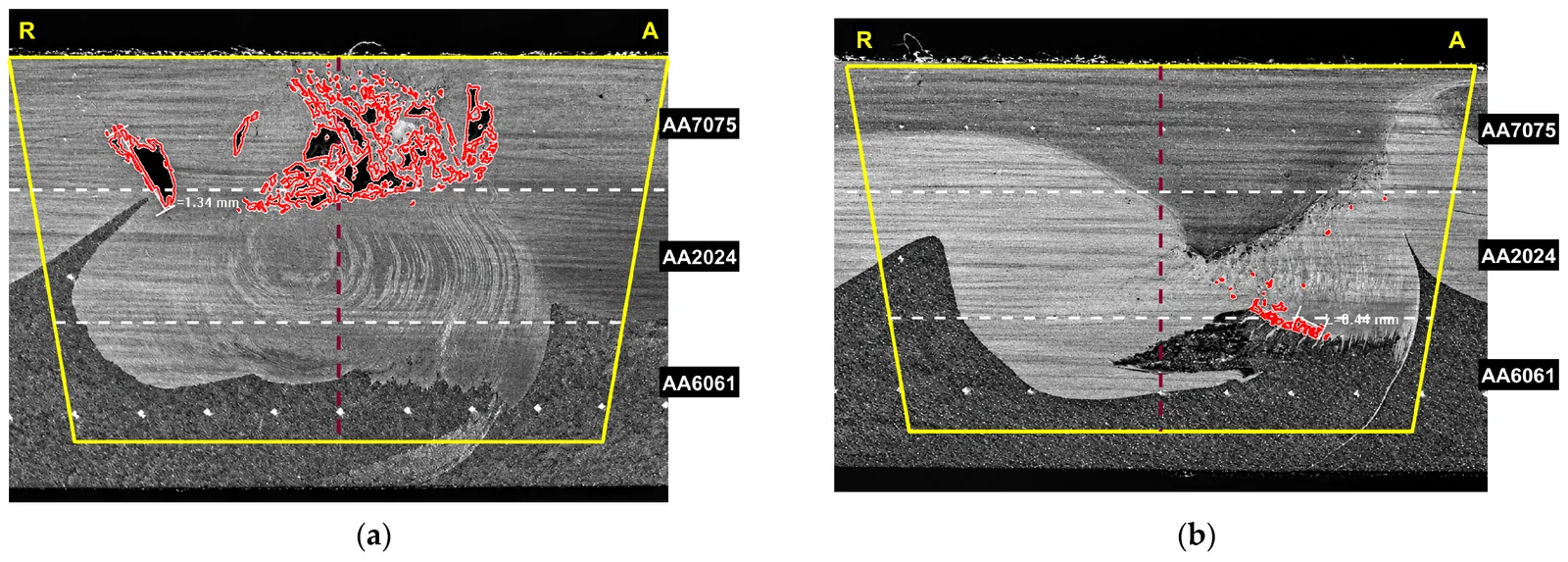
Spatial distribution of detected defect regions within the six analysis zones for two contrasting processing conditions: (**a**) Experiment 4.2 (1100 rpm, 60 mm/min) and (**b**) Experiment 4.3 (650 rpm, 100 mm/min).

**Figure 21:**
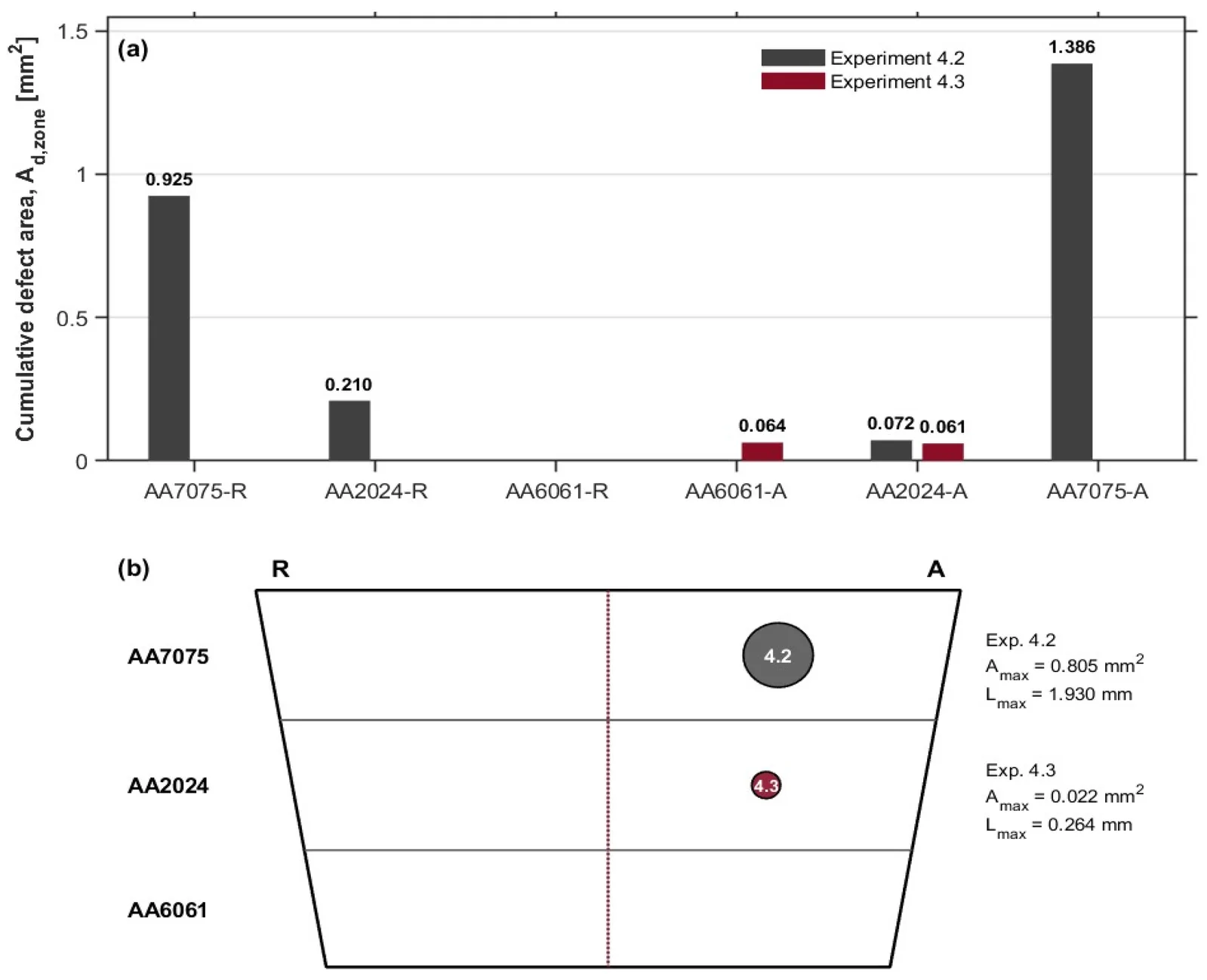
Zone-based distribution of defects for the contrasting processing conditions represented by Experiments 4.2 and 4.3: (**a**) cumulative defect area within the six analysis zones; (**b**) location and size of the largest detected defect within the multilayer cross-section.

**Figure 22:**
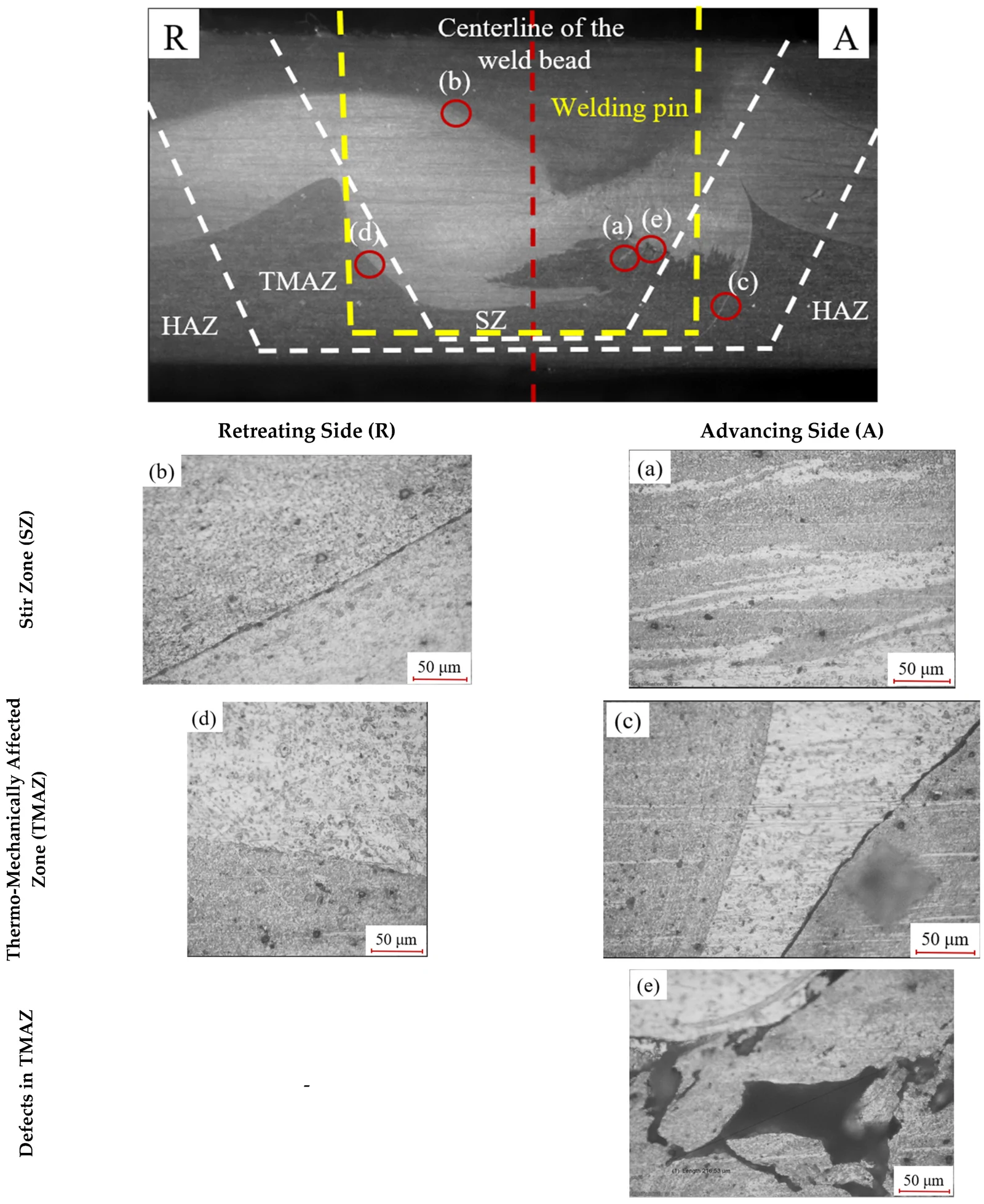
Microstructural features of the FSW joint obtained in Experiment 4.3 (650 rpm and 100 mm/min): (**a**,**b**) stir zone (SZ); (**c**,**d**) thermo-mechanically affected zone (TMAZ); and (**e**) defect observed in the TMAZ.

**Figure 23:**
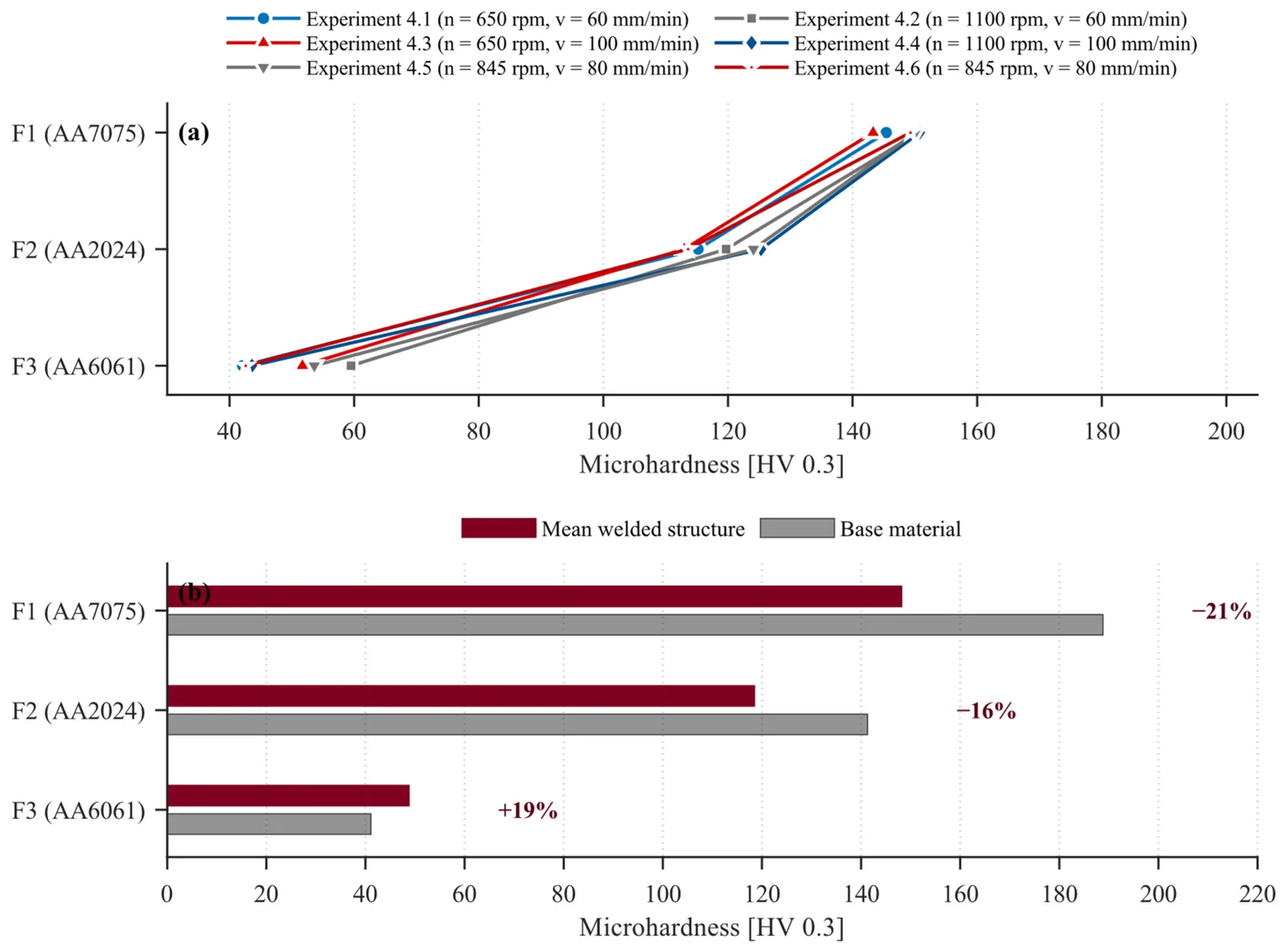
Average microhardness values as a function of the FSW process parameters: (**a**) microhardness values measured for the three layers (F1–F3) under the six experimental conditions; (**b**) comparison between the mean microhardness of the welded structure and the corresponding base material values, including the relative percentage variation.

**Table 1:** Chemical and mechanical properties of the aluminum alloys: AA2024-T3, AA6061-O and AA7075-T6.

Charact.	Chemical Composition [%]									Mechanical Characteristics		
	Si	Fe	Cu	Mn	Mg	Cr	Zn	Ti	Ti + Zr	Rm [MPa]	Rp0.2 [MPa]	At [%]
AA2024-T3	0.10	0.11	4.40	0.47	1.50	0.01	0.14	0.04	0.05	464–466	344–348.8	17–18
AA6061-O	0.74	0.40	0.22	0.14	0.90	0.18	0.09	0.05	-	117–121	63–69	24–29
AA7075-T6	0.05	0.10	1.60	0.05	2.70	0.19	5.80	0.05	0.06	593–594	531–532	11–12

**Table 2:** Design of Experiments for FSW process.

Stage	Exp. Code	Position of Aluminum Alloys	Tool Rotational Speed (rpm)	Welding Speed (mm/min)
Step 1	1	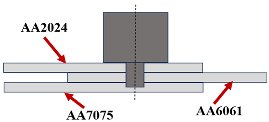	600	170
	2	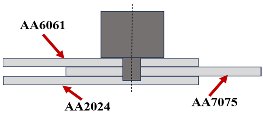		
	3	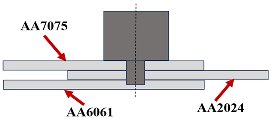		
Step 2	4.1	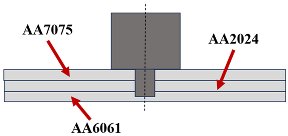	650	60
	4.2		1100	60
	4.3		650	100
	4.4		1100	100
	4.5		845	80
	4.6		845	80

**Table 3:** Average microhardness values of base materials.

Aluminum Alloy	AA2024	AA6061	AA7075
Microhardness [HV 0.3]	141.3	41.04	188.8

**Table 4:** Quantitative defect characteristics determined from the macrostructural cross-sections for the six second-stage experimental conditions.

Experiment	*n* [rpm]	*v* [mm/min]	*N_c_*	*A_d_* [mm^2^]	*f_A_* [%]	*A_max_* [mm^2^]	*L_max_* [mm]
4.1	650	60	59	0.926	1.774	0.194	1.086
4.2	1100	60	101	2.471	4.735	0.805	1.93
4.3	650	100	19	0.072	0.138	0.022	0.264
4.4	1100	100	100	2.285	4.378	0.649	1.763
4.5	845	80	67	1.686	3.230	0.525	1.762
4.6	845	80	40	1.454	2.785	1.124	2.268

## Data Availability

The raw data supporting the conclusions of this article will be made available by the authors on request.

## Abbreviations

The following abbreviations are used in this manuscript:

FSW	Friction stir welding
SZ	Stir zone
TMAZ	Thermo-mechanically affected zone
HAZ	Heat-affected zone
A	Advancing side
R	Retreating side
HF	Hydrofluoric acid
